# A meta-analysis of HLA peptidome composition in different hematological entities: entity-specific dividing lines and “pan-leukemia” antigens

**DOI:** 10.18632/oncotarget.14918

**Published:** 2017-01-31

**Authors:** Linus Backert, Daniel Johannes Kowalewski, Simon Walz, Heiko Schuster, Claudia Berlin, Marian Christoph Neidert, Mirle Schemionek, Tim H. Brümmendorf, Vladan Vucinic, Dietger Niederwieser, Lothar Kanz, Helmut Rainer Salih, Oliver Kohlbacher, Katja Weisel, Hans-Georg Rammensee, Stefan Stevanović, Juliane Sarah Walz

**Affiliations:** ^1^ Institute for Cell Biology, Department of Immunology, University of Tübingen, Tübingen, Germany; ^2^ Applied Bioinformatics, Center for Bioinformatics and Department of Computer Science, University of Tübingen, Tübingen, Germany; ^3^ Immatics Biotechnologies GmbH, Tübingen, Germany; ^4^ Department of Hematology and Oncology, University of Tübingen, Tübingen, Germany; ^5^ Department of Neurosurgery, University Hospital Zurich, University of Zurich, Zurich, Switzerland; ^6^ Department of Hematology, Oncology, Hemostaseology and SCT, University Hospital RWTH Aachen, Aachen, Germany; ^7^ University Hospital Leipzig, Department of Hematology and Oncology, Leipzig, Germany; ^8^ Clinical Cooperation Unit Translational Immunology, German Cancer Consortium (DKTK), DKFZ Partner Site Tübingen, Tübingen, Germany; ^9^ Quantitative Biology Center, University of Tübingen, Tübingen, Germany; ^10^ Biomolecular Interactions, Max Planck Institute for Developmental Biology, Tübingen, Germany; ^11^ German Cancer Consortium (DKTK), DKFZ Partner Site Tübingen, Tübingen, Germany

**Keywords:** hematological malignancies, cancer immunotherapy, tumor antigen, HLA, mass spectrometry

## Abstract

Hematological malignancies (HM) are highly amenable targets for immunotherapeutic intervention and may be effectively treated by antigen-specific T-cell based treatment. Recent studies demonstrate that physiologically occurring anti-cancer T-cell responses in certain HM entities target broadly presented non-mutated epitopes. HLA ligands are thus implied as prime targets for broadly applicable and antigen-specific off-the-shelf compounds. With the aim of assessing the presence of common targets shared among different HM which may enable addressing a larger patient collective we conducted a meta-analysis of 83 mass spectrometry-based HLA peptidome datasets (comprising 40,361 unique peptide identifications) across four major HM (19 AML, 16 CML, 35 CLL, and 13 MM/MCL samples) and investigated similarities and differences within the HLA presented antigenic landscape. We found the cancer HLA peptidome datasets to cluster specifically along entity and lineage lines, suggesting that the immunopeptidome directly reflects the differences in the underlying (tumor-)biology. In line with these findings, we only detected a small set of entity-spanning antigens, which were predominantly characterized by low presentation frequencies within the different patient cohorts. These findings suggest that design of T-cell immunotherapies for the treatment of HM should ideally be conducted in an entity-specific fashion.

## INTRODUCTION

In contrast to the recent breakthrough advances in the treatment of solid malignancies by antigen-unspecific immune-checkpoint blockade [1–6] the success of this highly promising treatment modality has so far been limited in hematological cancers [7, 8] with the prominent exception of Hodgkin lymphoma [9, 10]. As clinical effectiveness of checkpoint inhibition has been shown to be directly correlated to mutational load in solid tumors [11, 12] and mutation-derived neoepitopes have been identified as targets of the resultant anti-tumor T-cell responses [13–15], it may be surmised that the suboptimal effectiveness in hematologic malignancies (HM) may at least in part be attributed to the predominantly low mutational burden of these cancer entities [16, 17]. On the other hand, HM can be effectively treated by stem cell transplantation [18–20], donor lymphocyte infusion [21–23] or the more recently developed adoptive approaches utilizing chimeric antigen receptor (CAR) T cells, which showed breakthrough effectiveness, even in previously therapy-resistant forms of malignancy [24–26]. However, apart from the latter, these approaches are hampered by their infrequent effectiveness and, more importantly, severe off-target toxicity such as graft-versus-host disease. As CAR T-cell therapy likely will remain restricted to only a handful of cell surface (differentiation) antigens (e.g. CD19 [26], HER2 [27], CEA [28]), there is a pressing need to identify new targets and suitable treatment strategies for hematological malignancies not amenable to CAR T-cell therapy. For this aim, the identification of HLA-restricted T-cell epitopes on HM and their implementation in adoptive, engineering- or vaccine-based T-cell immunotherapy is a highly attractive option, rendering a vast array of intracellular - and potentially more specific - HM antigens amenable to immunological targeting. To this end our group and others have extensively studied the HLA-presented immunopeptidome of hematological cancers including acute myeloid leukemia (AML) [29], chronic myeloid leukemia (CML) [30], chronic lymphocytic leukemia (CLL) [31] and multiple myeloma (MM) [32], which led to the identification of multiple pathophysiologically relevant epitopes of anti-HM T-cell responses and inspired the notion that immune control in these low-mutational entities may effectively be mediated by T cells targeting non-mutated epitopes [33]. As the development of novel immunotherapeutic compounds is a highly cost- and time-intensive enterprise [34, 35], such non-mutant, common antigens represent highly attractive targets for off-the-shelf immunotherapy, which may be suited for the effective treatment of a substantial proportion of the patient population.

In this study we present a meta-analysis of our previous studies on the immunopeptidomes of the four major hematologic cancers in adults, AML [29], CML [30], CLL [31] and MM [32], addressing the similarity of these malignancies on the immunologically pivotal level of HLA-restricted presentation with the dedicated aim of investigating the existence and prevalence of potential “pan-leukemia antigens”.

## RESULTS

### Hierarchical clustering of HLA-restricted antigens on source protein level does not discern specific hematological malignancies

**Figure 1:**
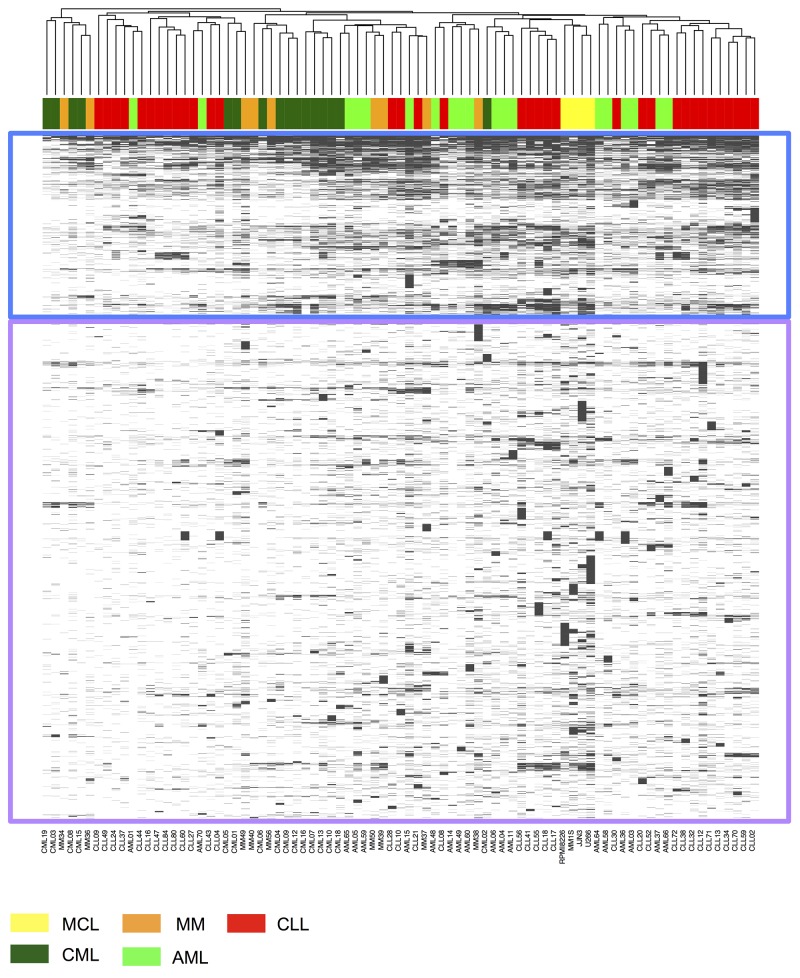
Unsupervised clustering analysis of HLA ligand source proteins represented in the immunopeptidomes of HM Peptides identified by LC-MS/MS in HLA class I ligand extracts of AML (light green, n=19), CML (dark green, n=16), CLL (red, n=35) and MM/MCL (orange/yellow, n=9/4) were mapped to their source proteins. For conserved sequences mapping to multiple proteins all protein annotations were retained. Complete linkage clustering was performed based on the Jaccard similarity coefficient of HLA ligand source proteins. A subset of source proteins shared across samples and entities with high frequencies of presentation is highlighted in the blue box; infrequent, sample/entitity-specific source proteins are highlighted in the purple box.

In order to obtain a comprehensive overview of the antigenic landscape of the four major HM and their immunological relatedness, we first performed an unsupervised hierarchical clustering of source proteins (8,053 unique source proteins) represented by HLA-restricted peptides (40,361 unique peptide IDs) in the immunopeptidomes of primary AML (n=19), CML (n=16), CLL (n=35) and MM/myeloma cell lines (MCL) (n=9/4, Figure 1). Without stratification of patients for expression of specific HLA allotypes, this source protein level analysis did not delineate clusters along entity lines but rather revealed that the antigenic landscape is divided into a smaller subset of non-entity specific common antigens (Figure 1, upper box) juxtaposed with a larger, highly heterogeneous set of sample-specific antigens (Figure 1, lower box). Whereas the larger group of sample and subset-specific source proteins clearly reflects a high degree of tumor/patient individuality, the presence of a smaller common subset of antigens hints at the potential presence of highly frequent and entity-spanning pan-leukemia antigens. In order to evaluate the presence of such targets in the HM dataset, we shifted our analysis to the HLA peptidome level, specifically filtered for HLA ligands which were exclusively detected on tumor tissue and subsequently performed HLA allotype-specific immunopeptidome profiling and cluster analyses for the seven most common HLA allotypes (A*01:01, A*02:01, A*03:01, A*24:02, B*07:02, B*08:01, B*18:01; >95% population coverage in the Caucasian population) [36]. To this end, we first subtracted from the dataset of HM-derived HLA ligands any peptide (irrespective of HLA restriction) also contained in our comprehensive in-house database of HLA ligands detected on non-malignant primary tissue specimens (n=147, number of unique peptides: 44,541, Supplementary Table 1). For the remaining set of peptides, which were only detected on malignant samples (from now on referred to as “cancer-exclusive”) we computationally assigned the restricting HLA allotypes and compiled allotype-specific HLA ligand datasets for further analysis (Supplementary Table 2).

Importantly, we cannot rule out presentation of these “cancer-exclusive” HLA ligands on normal (sub-)tissues or cell populations at levels below the limit of the detection or on sample types missing in our normal tissue database.

### HM entities and lineages can be distinguished purely based on HLA allotype-specific immunopeptidome composition

**Figure 2:**
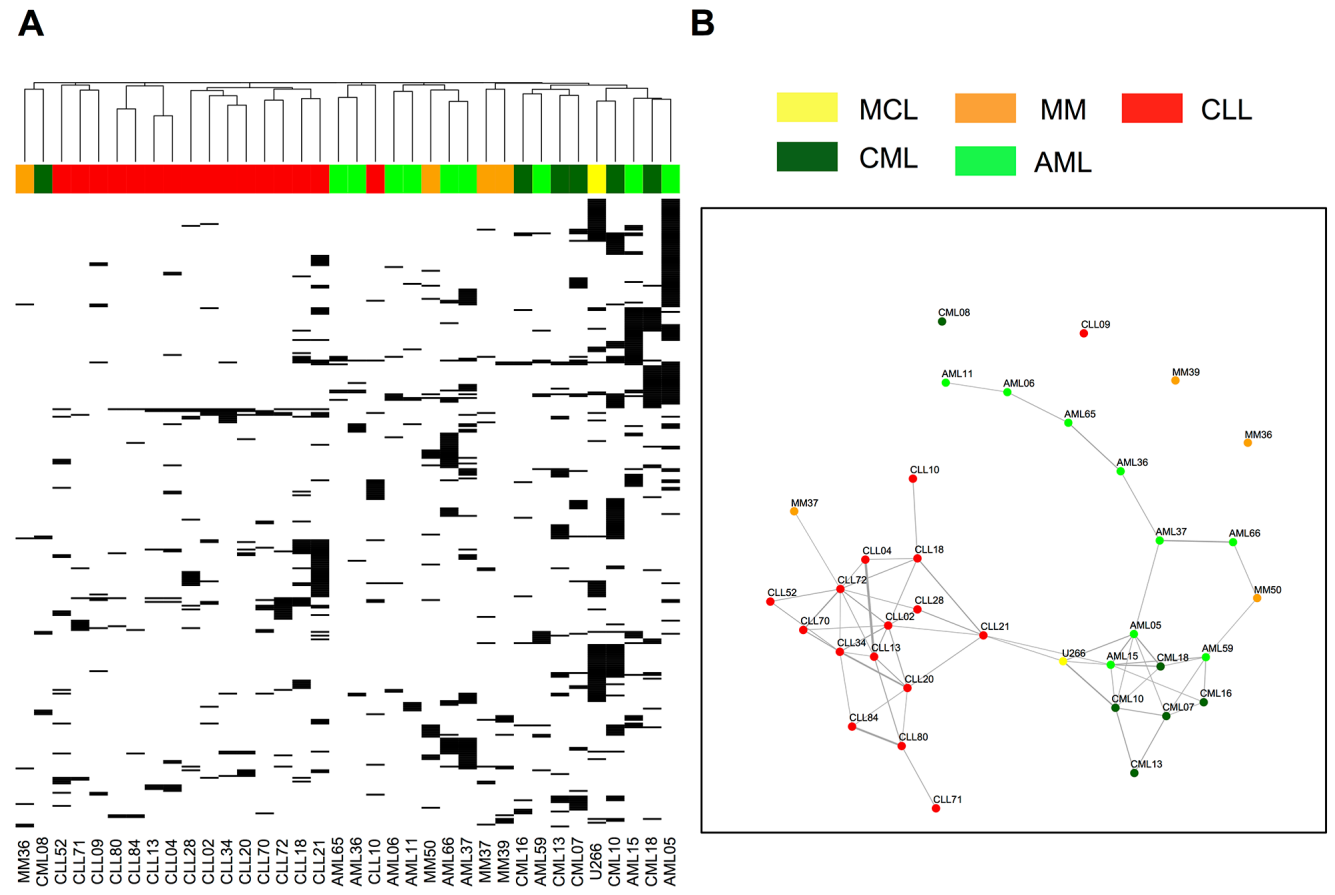
Unsupervised clustering analysis and Jaccard distance graphs of “cancer-exclusive” HLA-A*02:01 ligands on hematological cancers “Cancer-exclusive” HLA-A*02:01 ligands identified on AML (light green, n=9), CML (dark green, n=6), CLL (red, n=16) and MM/MCL (orange/yellow, n=4/1) were analyzed by: **A**. Complete linkage clustering based on the Jaccard similarity coefficient of A*02:01 immunopeptidomes. **B**. Jaccard distance graphs. Samples showing ≥10% Jaccard similarity of their “cancer-exclusive” HLA-A*02:01 immunopeptidomes were linked by edges, with the thickness of the edge positively correlating with the degree of similarity.

Unsupervised clustering analysis of the HLA-A*02:01-restricted HM immunopeptidomes (AML (n=9), CML (n=6), CLL (n=16), MM/MCL (n=4/1)) resulted in clear clustering of samples belonging to the same hematological cancer entities, as well as coherent clustering of the lineages these malignancies arise from (Figure 2A). This suggests that the HLA ligandome directly reflects tumor/lineage-specific biology, which is further underscored by the findings of gene ontology analyses (GO Term BP) using DAVID [37], which identified B-cell receptor signaling (GO ID: 0050853) as a significantly enriched biological process (*P*<0.05 after Benjamini-Hochberg correction for multiple testing) represented selectively in the immunopeptidome of the lymphatic lineage (CLL and MM/MCL).

To further investigate and visualize the inter-relatedness of samples and to assess lineage-specific dividing lines, we performed Jaccard distance graph analysis, which identified sub-networks of closely related (≥10% immunopeptidome overlap, linked by edges) CLL and CML samples. AML on the other hand showed a longitudinally interlinked chain of related samples, which covers a vast range of possible A*02:01 immunopeptidome compositions. Connections across entity boundaries were only identified in two isolated cases (Figure 2B). For the other HLA allotypes similar observations were made, with clear entity-specific dividing lines detected in all cases and CLL universally clustering in centralized subnetworks (Supplementary Figures 1 and 2).

Together, these findings suggest that peptide-specific T-cell immunotherapy in hematologic malignancies may have to be designed in an entity-specific fashion. However, it has to be noted that the underlying analysis was selectively implemented to assess similarities in immunopeptidome composition–and, by proxy, tumor biology—and does not provide the sensitivity to detect individual shared pan-leukemia antigens. To specifically achieve this goal and evaluate the potential presence of broadly applicable targets for off-the-shelf immunotherapy of multiple HM with a single compound, we further sought for individual, shared HLA ligands.

### Overlap analysis identifies a small panel of naturally presented “pan-leukemia” antigens

**Figure 3:**
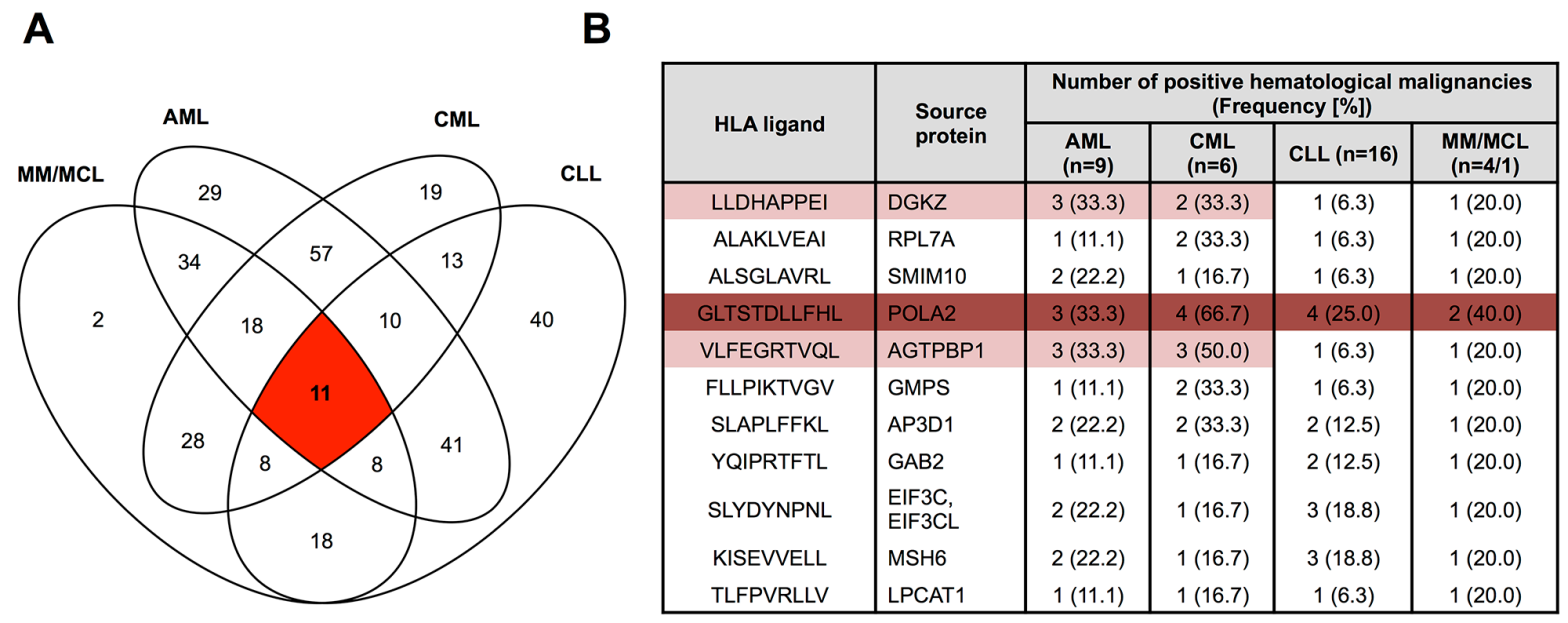
Presentation of “cancer”-exclusive HLA-A*02:01 ligands across different hematological malignancies **A**. Overlap analysis of “cancer-exclusive” HLA-A*02:01 ligands identified on AML (n=9), CML (n=6), CLL (n=16) and MM/MCL (n=4/1). **B**. HLA-A*02:01 restricted “pan-leukemia” antigens identified across all four hematological malignancies. Peptides represented with frequencies ≥20% across all entities are marked in dark red, peptides represented with frequencies ≥20% across entities of the same lineage are marked in light red. A minimum value of n≥4 allotype positive samples was required for the calculation of presentation frequencies.

Allotype-specific overlap analysis of HM-derived HLA ligands identified 25 unique HLA ligands (A*01:01: 0; A*02:01: 11; A*03:01: 9; A*24:02: 0; B*07:02: 2; B*08:01: 0; B*18:01: 3) showing “cancer-exclusive” presentation on all four HM simultaneously (Figure 3A, Supplementary Tables 3–5, Supplementary Figure 3). Thus, universal antigen presentation across entity boundaries is a very rare phenomenon, which is further aggravated by the fact that shared antigens typically show low presentation frequencies within the different HM entities (Figure 3B). A single pan-leukemia peptide with presentation frequency of more than 20% across all entities was identified for HLA-A*02:01 (POLA2_470-480_, GLTSTDLLFHL). Furthermore, lineage-specific analysis highlights three myeloid lineage-specific antigens and six lymphatic lineage-specific antigens with presentation frequencies above 20% (with a minimum value of n≥4 allotype positive samples applied for the calculation of presentation frequencies, Figure 3B, Supplementary Table 3–5). However, these targets were so far not evaluated for immunogenicity or tumor-specific cytotoxicity. Together, these results clearly argue in favor of entity-specific antigen discovery for T cell immunotherapy in HM, albeit our analysis identified very few novel, broadly presented candidate targets, which may be amenable for further drug development.

## DISCUSSION

In the wake of the clinical success of immune checkpoint modulation, it became more and more evident that novel, supplementary therapeutic interventions may be required for a range of malignancies and patient collectives showing low response rates to checkpoint inhibitor monotherapy [11, 38]. For this reason therapeutic strategies aimed at inducing antigen-specific anti-tumor T-cell responses have experienced a surge of renewed interest [39]. Common prerequisite to all these approaches is the exact knowledge of clinically effective targets specifically presented on HLA molecules on malignant cells. While the current paradigm views mutation-derived neoepitopes as the most highly effective targets of anti-tumor T-cell responses [12, 40, 41], this mutation-centric view severely limits the range of malignancies deemed eligible for T-cell immunotherapy [16, 17]. Furthermore, mutation-specific strategies would, at least in most cases, be patient-individualized and thus require massively time-and cost intensive target discovery and validation, which currently poses a severe limitation to the number of patients eligible for such approaches^34,35^. Together, these circumstances prompted us and others to comprehensively investigate the non-mutant antigenic landscape presented by HLA molecules on different low-mutational cancer entities [42–47]. Importantly, our previous studies in hematological malignancies demonstrated that 1) vast arrays of non-mutated but nevertheless cancer-specific HLA ligands are presented on these cancers, which may be explained by altered antigen processing in malignant cells [33] 2) these peptides are immunogenic and targeted by physiologically occurring T-cell responses in patients [29, 32] and 3) that anti-leukemia T-cell responses do correlate with improved patient survival in CLL patients underlining their central role in cancer immune control [31].

Based on these studies we herein conducted a meta-analysis aimed at assessing the particularities of four major HM on the immunopeptidome level and gauged the possibility of identifying a set of universal “pan-leukemia” antigens. In order to evaluate whether the immunopeptidome directly reflects the different biology of the four HM, we assessed the relatedness of all samples on the HLA ligand source protein level. This did not result in grouping of samples according to their respective entities but revealed the existence of a common set of “housekeeping” antigens represented across all entities. This was expected based on our previous studies and is in line with findings of another study on the immunopeptidome of cell lines derived from different tissue origins [48].

Even though our analysis was aimed at removing the impact of different HLA types from the equation by clustering on the level of HLA ligand source proteins, a pattern of HLA allotype-dependent selection for specific source proteins is clearly evident when comparing this cluster analysis with the results of HLA allotype-stratified clustering of HM ligands. Where source protein clustering did not result in coherent grouping of samples along entity lines, the allotype-specific analysis clearly delineated samples according to their entity and lineage of origin, indicating a major influence of sample HLA types on protein representation in the immunopeptidome. Importantly, robust clustering of entity and lineage subgroups was observed for all seven HLA allotypes analyzed in this study. This underscores the robustness of our analytical pipeline and demonstrates that tumor- and lineage specific biology is reflected in the HLA peptidome, which points to the possibility of confidently identifying and assigning pathology purely based on immunopeptidome data (an approach which was previously presented for proteomics data [49]). On the other hand, this finding hints at the limited occurrence of broadly shared antigens, which led us to employ simple overlap analysis as a sensitive means to identify this sparse population of entity-spanning HLA ligands. This verified the rarity of “pan-leukemia” antigens, as such peptides were only detectable for four out of seven HLA allotypes and furthermore typically showed only low frequencies of presentation within the different entities. None of these “pan-leukemia” antigens derives from established tumor-associated genes, which may be explained by a distorted correlation of gene expression and HLA restricted antigen presentation and underscores the importance of direct antigen discovery by mass-spectrometry [48, 50]. However –importantly- it also has to be noted that several factors pose central challenges for mass spectrometry based antigen discovery: limited sensitivity and dynamic range as well as the stochasticity of sampling in data-dependent mass spectrometry may lead to false-positive tumor-exclusive detection.

Our central finding is the presence of entity- and lineage-specific dividing lines, which may vitally impede the development of entity-spanning antigen-specific compounds. This strongly argues in favor of entity-specific approaches for the development of antigen-specific T-cell immunotherapy in hematological malignancies.

## MATERIALS AND METHODS

### Patient blood and bone marrow samples

Peripheral mononuclear cells (PBMC) from AML, CLL and CML patients and bone marrow mononuclear cells (BMNC) from MM patients (provided by the Departments of Hematology and Oncology in Tübingen, Leipzig and Aachen, Germany) at the time of initial diagnosis or relapse prior to therapy were isolated by density gradient centrifugation (Biocoll, Biochrom GmbH, Berlin, Germany) and erythrocyte lysis (EL buffer, Qiagen, Venlo, Netherlands). For all AML and CLL samples the frequency of malignant cells within the PBMC isolate was > 80%. For MM samples the percentage of malignant plasma cells within the BMNC fraction was > 60%. For CML we analyzed whole blood samples of 12 CML patients in the chronic phase (no blasts), two in the accelerated phase (18-20% myeloid blasts) and two in a blast crisis (50-60% myeloid blasts). Informed consent was obtained in accordance with the Declaration of Helsinki protocol. The study was performed according to the guidelines of the local ethics committee (373/2011BO2, 142/2013BO2). HLA typing was carried out by the Department of Hematology and Oncology, Tübingen, Germany. Samples were stored at −80 °C until further use.

### Healthy control tissue samples

PBMC and bone marrow mononuclear cells (BMNC) from healthy volunteers were isolated by density gradient centrifugation (Biocoll, Biochrom GmbH, Berlin, Germany) and erythrocyte lysis (EL buffer, Qiagen, Venlo, Netherlands). Normal tissue samples from patients and autopsy material were provided by the University Hospital Tübingen, Germany and the University Hospital Zürich, Switzerland (Supplementary Table 1). Specimens were frozen in liquid nitrogen immediately after resection. Informed consent was obtained in accordance with the Declaration of Helsinki protocol

### Myeloma cell lines (MCL)

For HLA ligandome analysis the myeloma cell lines (MCLs, U266, RPMI8226 and JJN3) were cultured in the recommended cell media (RPMI1640 (Gibco, Carlsbad, CA, USA), IMDM (Lonza, Basel, Switzerland)) supplemented with fetal calf serum, 100 IU/L penicillin, 100 mg/L streptomycin, and 2 mmol/L glutamine at 37°C and 5% CO_2_.

### Isolation of HLA ligands from primary samples and MCLs

HLA class I molecules were isolated using standard immunoaffinity purification as described before [29, 51], using the pan-HLA class I specific mAb W6/32 (produced in house) to extract HLA ligands.

### Analysis of HLA ligands by LC-MS/MS

HLA ligand extracts were analyzed in five technical replicates as described previously [31]. In brief, peptide samples were separated by nanoflow HPLC (RSLCnano, Thermo Fisher, Waltham, MA, USA) using a 50 μm×25 cm PepMap RSLC column (Thermo Fisher) and a gradient ranging from 2.4 to 32.0% acetonitrile over the course of 90 min. Eluting peptides were analyzed in an online-coupled LTQ Orbitrap XL mass spectrometer (Thermo Fisher) using a top 5 CID (collision-induced dissociation) fragmentation method.

### Database search and HLA annotation

Data processing was performed as described previously [31]. In brief, the Mascot search engine (Mascot 2.2.04; Matrix Science, London, UK) was used to search the human proteome as comprised in the Swiss-Prot database (20,279 reviewed protein sequences, September 27^th^, 2013) without enzymatic restriction. Oxidized methionine was allowed as a dynamic modification. The peptide-level false discovery rate (FDR) was estimated using the Percolator algorithm (v2.04) [52] and set to 5%. Peptide lengths were limited to 8-12 amino acids. Protein inference was disabled, allowing for multiple protein annotations of peptides. HLA annotation was performed using NetMHCpan (v3.0) [53], annotating peptides with IC_50_ scores ≤ 500 nM and/or percentile ranks ≤ 2% as ligands of the corresponding HLA allotype. Samples for which only two-digit HLA typings were available, the missing sub-alleles were inferred based on the assumption of the most frequent four-digit allotype. For quality control, yield thresholds of ≥200 unique HLA class I ligands for primary samples and ≥1,000 unique HLA class I ligands for MCL were applied.

### Software and statistical analysis

Data processing and analysis was performed in Python 2.7.10 and R 3.3.1 [54]. The heat maps, Jaccard index graphs, and Venn diagrams were created using the R packages gplots [55], igraph [56], and VennDiagram [57]. The clustering and distance graph analysis was performed using complete linkage clustering and the Jaccard distance, which measures the qualitative dissimilarity between two HLA peptidomes. The Jaccard distance in these analyses was calculated as the difference of the size of the union and the intersection of HLA peptidomes divided by the size of their union in pairwise comparison for all possible sample combinations. This metric was selected as it is equivalent to overlap visualization by Venn diagrams commonly utilized in HLA peptidomics studies. Thresholds were defined empirically, with Jaccard similarities of 0.1 yielding optimal sensitivity and specificity. For clustering, Jaccard distance graphs and overlap analysis of “cancer-exclusive” HLA ligand datasets obtained after subtraction of the normal tissue peptidome, HLA ligands occurring only once across all samples were discarded and only samples containing more than five unique HLA class I ligands were included.

## SUPPLEMENTARY MATERIALS FIGURES AND TABLES





## References

[1] Couzin-Frankel J (2013). Breakthrough of the year 2013. Cancer immunotherapy. Science.

[2] McDermott DF, Drake CG, Sznol M, Choueiri TK, Powderly JD, Smith DC, Brahmer JR, Carvajal RD, Hammers HJ, Puzanov I, Hodi FS, Kluger HM, Topalian SL (2015). Survival, durable response, and long-term safety in patients with previously treated advanced renal cell carcinoma receiving nivolumab. J Clin Oncol.

[3] Brahmer J, Reckamp KL, Baas P, Crino L, Eberhardt WE, Poddubskaya E, Antonia S, Pluzanski A, Vokes EE, Holgado E, Waterhouse D, Ready N, Gainor J (2015). Nivolumab versus docetaxel in advanced squamous-cell non-small-cell lung cancer. N Engl J Med.

[4] Gettinger SN, Horn L, Gandhi L, Spigel DR, Antonia SJ, Rizvi NA, Powderly JD, Heist RS, Carvajal RD, Jackman DM, Sequist LV, Smith DC, Leming P (2015). Overall survival and long-term safety of nivolumab (Anti-Programmed Death 1 Antibody, BMS-936558, ONO-4538) in patients with previously treated advanced non-small-cell lung cancer. J Clin Oncol.

[5] Riley JL (2013). Combination checkpoint blockade--taking melanoma immunotherapy to the next level. N Engl J Med.

[6] Motzer RJ, Escudier B, McDermott DF, George S, Hammers HJ, Srinivas S, Tykodi SS, Sosman JA, Procopio G, Plimack ER, Castellano D, Choueiri TK, Gurney H (2015). Nivolumab versus everolimus in advanced renal-cell carcinoma. N Engl J Med.

[7] Lesokhin AM, Ansell SM, Armand P, Scott EC, Halwani A, Gutierrez M, Millenson MM, Cohen AD, Schuster SJ, Lebovic D, Dhodapkar MV, Avigan D, Chapuy B (2014). Preliminary results of a phase i study of nivolumab (BMS-936558) in patients with relapsed or refractory lymphoid malignancies. Blood ASH Abstr.

[8] Ansell SM, Hurvitz SA, Koenig PA, LaPlant BR, Kabat BF, Fernando D, Habermann TM, Inwards DJ, Verma M, Yamada R, Erlichman C, Lowy I, Timmerman JM (2009). Phase I study of ipilimumab, an anti-CTLA-4 monoclonal antibody, in patients with relapsed and refractory B-cell non-Hodgkin lymphoma. Clin Cancer Res.

[9] Armand P, Shipp MA, Ribrag V, Michot JM, Zinzani PL, Kuruvilla J, Snyder ES, Ricart AD, Balakumaran A, Rose S, Moskowitz CH (2016). Programmed death-1 blockade with pembrolizumab in patients with classical hodgkin lymphoma after brentuximab vedotin failure. J Clin Oncol.

[10] Ansell SM, Lesokhin AM, Borrello I, Halwani A, Scott EC, Gutierrez M, Schuster SJ, Millenson MM, Cattry D, Freeman GJ, Rodig SJ, Chapuy B, Ligon AH (2015). PD-1 blockade with nivolumab in relapsed or refractory Hodgkin’s lymphoma. N Engl J Med.

[11] Rizvi NA, Hellmann MD, Snyder A, Kvistborg P, Makarov V, Havel JJ, Lee W, Yuan J, Wong P, Ho TS, Miller ML, Rekhtman N, Moreira AL, Cancer immunology (2015). Mutational landscape determines sensitivity to PD-1 blockade in non-small cell lung cancer. Science.

[12] Snyder A, Makarov V, Merghoub T, Yuan J, Zaretsky JM, Desrichard A, Walsh LA, Postow MA, Wong P, Ho TS, Hollmann TJ, Bruggeman C, Kannan K (2014). Genetic basis for clinical response to CTLA-4 blockade in melanoma. N Engl J Med.

[13] Gubin MM, Zhang X, Schuster H, Caron E, Ward JP, Noguchi T, Ivanova Y, Hundal J, Arthur CD, Krebber WJ, Mulder GE, Toebes M, Vesely MD (2014). Checkpoint blockade cancer immunotherapy targets tumour-specific mutant antigens. Nature.

[14] Gros A, Parkhurst MR, Tran E, Pasetto A, Robbins PF, Ilyas S, Prickett TD, Gartner JJ, Crystal JS, Roberts IM, Trebska-McGowan K, Wunderlich JR, Yang JC, Rosenberg SA (2016). Prospective identification of neoantigen-specific lymphocytes in the peripheral blood of melanoma patients. Nat Med.

[15] Stronen E, Toebes M, Kelderman S, van Buuren MM, Yang W, van Rooij N, Donia M, Boschen ML, Lund-Johansen F, Olweus J, Schumacher TN (2016). Targeting of cancer neoantigens with donor-derived T cell receptor repertoires. Science.

[16] Alexandrov LB, Nik-Zainal S, Wedge DC, Aparicio SA, Behjati S, Biankin AV, Bignell GR, Bolli N, Borg A, Borresen-Dale AL, Boyault S, Burkhardt B, Butler AP (2013). Signatures of mutational processes in human cancer. Nature.

[17] Vogelstein B, Papadopoulos N, Velculescu VE, Zhou S, Diaz LA, Kinzler KW (2013). Cancer genome landscapes. Science.

[18] Weiden PL, Flournoy N, Thomas ED, Prentice R, Fefer A, Buckner CD, Storb R (1979). Antileukemic effect of graft-versus-host disease in human recipients of allogeneic-marrow grafts. N Engl J Med.

[19] Bleakley M, Riddell SR (2011). Exploiting T cells specific for human minor histocompatibility antigens for therapy of leukemia. Immunol Cell Biol.

[20] Ritgen M, Stilgenbauer S, von Neuhoff N, Humpe A, Bruggemann M, Pott C, Raff T, Krober A, Bunjes D, Schlenk R, Schmitz N, Dohner H, Kneba M, Dreger P (2004). Graft-versus-leukemia activity may overcome therapeutic resistance of chronic lymphocytic leukemia with unmutated immunoglobulin variable heavy-chain gene status: implications of minimal residual disease measurement with quantitative PCR. Blood.

[21] Russell NH, Byrne JL, Faulkner RD, Gilyead M, Das-Gupta EP, Haynes AP (2005). Donor lymphocyte infusions can result in sustained remissions in patients with residual or relapsed lymphoid malignancy following allogeneic haemopoietic stem cell transplantation. Bone Marrow Transplant.

[22] Roush KS, Hillyer CD (2002). Donor lymphocyte infusion therapy. Transfus Med Rev.

[23] Schmid C, Labopin M, Nagler A, Bornhauser M, Finke J, Fassas A, Volin L, Gurman G, Maertens J, Bordigoni P, Holler E, Ehninger G, Polge E (2007). Donor lymphocyte infusion in the treatment of first hematological relapse after allogeneic stem-cell transplantation in adults with acute myeloid leukemia: a retrospective risk factors analysis and comparison with other strategies by the EBMT Acute Leukemia Working Party. J Clin Oncol.

[24] Porter DL, Hwang WT, Frey NV, Lacey SF, Shaw PA, Loren AW, Bagg A, Marcucci KT, Shen A, Gonzalez V, Ambrose D, Grupp SA, Chew A (2015). Chimeric antigen receptor T cells persist and induce sustained remissions in relapsed refractory chronic lymphocytic leukemia. Sci Transl Med.

[25] Garfall AL, Maus MV, Hwang WT, Lacey SF, Mahnke YD, Melenhorst JJ, Zheng Z, Vogl DT, Cohen AD, Weiss BM, Dengel K, Kerr ND, Bagg A (2015). Chimeric antigen receptor T cells against cd19 for multiple myeloma. N Engl J Med.

[26] Maude SL, Frey N, Shaw PA, Aplenc R, Barrett DM, Bunin NJ, Chew A, Gonzalez VE, Zheng Z, Lacey SF, Mahnke YD, Melenhorst JJ, Rheingold SR (2014). Chimeric antigen receptor T cells for sustained remissions in leukemia. N Engl J Med.

[27] Zhao Y, Moon E, Carpenito C, Paulos CM, Liu X, Brennan AL, Chew A, Carroll RG, Scholler J, Levine BL, Albelda SM, June CH (2010). Multiple injections of electroporated autologous T cells expressing a chimeric antigen receptor mediate regression of human disseminated tumor. Cancer Res.

[28] Guest RD, Kirillova N, Mowbray S, Gornall H, Rothwell DG, Cheadle EJ, Austin E, Smith K, Watt SM, Kuhlcke K, Westwood N, Thistlethwaite F, Hawkins RE, Gilham DE (2014). Definition and application of good manufacturing process-compliant production of CEA-specific chimeric antigen receptor expressing T-cells for phase I/II clinical trial. Cancer Immunol Immunother.

[29] Berlin C, Kowalewski DJ, Schuster H, Mirza N, Walz S, Handel M, Schmid-Horch B, Salih HR, Kanz L, Rammensee HG, Stevanovic S, Stickel JS (2015). Mapping the HLA ligandome landscape of acute myeloid leukemia: a targeted approach toward peptide-based immunotherapy. Leukemia.

[30] Stickel JS, Kowalewski DJ, Berlin C, Schemionek M, Kanz L, Salih HR, Brümmendorf TH, Rammensee HG, Stevanovic S (2013). HLA ligandome analysis of chronic myeloid leukemia (CML), revealed novel tumor associated antigens for peptide based immunotherapy. Blood ASH Abstr.

[31] Kowalewski DJ, Schuster H, Backert L, Berlin C, Kahn S, Kanz L, Salih HR, Rammensee HG, Stevanovic S, Stickel JS (2015). HLA ligandome analysis identifies the underlying specificities of spontaneous antileukemia immune responses in chronic lymphocytic leukemia (CLL). Proc Natl Acad Sci U S A.

[32] Walz S, Stickel JS, Kowalewski DJ, Schuster H, Weisel K, Backert L, Kahn S, Nelde A, Stroh T, Handel M, Kohlbacher O, Kanz L, Salih HR (2015). The antigenic landscape of multiple myeloma: mass spectrometry (re)defines targets for T-cellbased immunotherapy. Blood.

[33] Kowalewski DJ, Stevanovic S, Rammensee HG, Stickel JS (2015). Antileukemia T-cell responses in CLL: we don’t need no aberration. Oncoimmunology.

[34] Walker A, Johnson R (2016). Commercialization of cellular immunotherapies for cancer. Biochem Soc Trans.

[35] Wang X, Riviere I (2015). Manufacture of tumor- and virus-specific T lymphocytes for adoptive cell therapies. Cancer Gene Ther.

[36] Schipper RF, D’Amaro J, Bakker JT, Bakker J, van Rood JJ, Oudshoorn M (1997). HLA gene haplotype frequencies in bone marrow donors worldwide registries. Hum Immunol.

[37] W Huang da, Sherman BT, Lempicki RA (2009). Systematic and integrative analysis of large gene lists using DAVID bioinformatics resources. Nat Protoc.

[38] Le DT Uram JN, Wang H, Bartlett BR, Kemberling H, Eyring AD, Skora AD, Luber BS, Azad NS, Laheru D, Biedrzycki B, Donehower RC, Zaheer A (2015). PD-1 blockade in tumors with mismatch-repair deficiency. N Engl J Med.

[39] Perez-Gracia JL, Labiano S, Rodriguez-Ruiz ME, Sanmamed MF, Melero I (2014). Orchestrating immune check-point blockade for cancer immunotherapy in combinations. Curr Opin Immunol.

[40] Robbins PF, Lu YC, El-Gamil M, Li YF, Gross C, Gartner J, Lin JC, Teer JK, Cliften P, Tycksen E, Samuels Y, Rosenberg SA (2013). Mining exomic sequencing data to identify mutated antigens recognized by adoptively transferred tumor-reactive T cells. Nat Med.

[41] van Rooij N, van Buuren MM, Philips D, Velds A, Toebes M, Heemskerk B, van Dijk LJ, Behjati S, Hilkmann H, El Atmioui D, Nieuwland M, Stratton MR, Kerkhoven RM (2013). Tumor exome analysis reveals neoantigen-specific T-cell reactivity in an ipilimumab-responsive melanoma. J Clin Oncol.

[42] Walter S, Weinschenk T, Stenzl A, Zdrojowy R, Pluzanska A, Szczylik C, Staehler M, Brugger W, Dietrich PY, Mendrzyk R, Hilf N, Schoor O, Fritsche J (2012). Multipeptide immune response to cancer vaccine IMA901 after single-dose cyclophosphamide associates with longer patient survival. Nat Med.

[43] Dutoit V, Herold-Mende C, Hilf N, Schoor O, Beckhove P, Bucher J, Dorsch K, Flohr S, Fritsche J, Lewandrowski P, Lohr J, Rammensee HG, Stevanovic S (2012). Exploiting the glioblastoma peptidome to discover novel tumour-associated antigens for immunotherapy. Brain.

[44] Bassani-Sternberg M, Barnea E, Beer I, Avivi I, Katz T, Admon A (2010). Soluble plasma HLA peptidome as a potential source for cancer biomarkers. Proc Natl Acad Sci U S A.

[45] Shraibman B, Melamed Kadosh D, Barnea E, Admon A (2016). HLA peptides derived from tumor antigens induced by inhibition of DNA methylation for development of drug-facilitated immunotherapy. Mol Cell Proteomics.

[46] Neumann A, Horzer H, Hillen N, Klingel K, Schmid-Horch B, Buhring HJ, Rammensee HG, Aebert H, Stevanovic S (2013). Identification of HLA ligands and T-cell epitopes for immunotherapy of lung cancer. Cancer Immunol Immunother.

[47] Stickel JS, Weinzierl AO, Hillen N, Drews O, Schuler MM, Hennenlotter J, Wernet D, Muller CA, Stenzl A, Rammensee HG, Stevanovic S (2009). HLA ligand profiles of primary renal cell carcinoma maintained in metastases. Cancer Immunol Immunother.

[48] Bassani-Sternberg M, Pletscher-Frankild S, Jensen LJ, Mann M (2015). Mass spectrometry of human leukocyte antigen class I peptidomes reveals strong effects of protein abundance and turnover on antigen presentation. Mol Cell Proteomics.

[49] Balog J, Sasi-Szabo L, Kinross J, Lewis MR, Muirhead LJ, Veselkov K, Mirnezami R, Dezso B, Damjanovich L, Darzi A, Nicholson JK, Takats Z (2013). Intraoperative tissue identification using rapid evaporative ionization mass spectrometry. Sci Transl Med.

[50] Weinzierl AO, Lemmel C, Schoor O, Muller M, Kruger T, Wernet D, Hennenlotter J, Stenzl A, Klingel K, Rammensee HG, Stevanovic S (2007). Distorted relation between mRNA copy number and corresponding major histocompatibility complex ligand density on the cell surface. Mol Cell Proteomics.

[51] Kowalewski DJ, Stevanovic S (2013). Biochemical large-scale identification of MHC class I ligands. Methods Mol Biol.

[52] Kall L, Canterbury JD, Weston J, Noble WS, MacCoss MJ (2007). Semi-supervised learning for peptide identification from shotgun proteomics datasets. Nat Methods.

[53] Nielsen M, Andreatta M (2016). NetMHCpan-3.0; improved prediction of binding to MHC class I molecules integrating information from multiple receptor and peptide length datasets. Genome Med.

[54] Team RC (2016). R: A language and environment for statistical computing. R Foundation for Statistical Computing.

[55] Warnes GR, Bolker B, Bonebakker L, Gentleman R, Andy Liaw WH, Lumley T, Maechler M, Magnusson A, Moeller S, Schwartz M, Venables B (2016). gplots: various R programming tools for plotting data.

[56] Csardi G, Nepusz T (2006). The igraph software package for complex network research, InterJournal, Complex Systems 1695.

[57] Chen H VennDiagram: generate high-resolution Venn and Euler plots.

