# Supplementary material for: A meta-analysis of HLA peptidome composition in different hematological entities: entity-specific dividing lines and “pan-leukemia” antigens
Source: Oncotarget. 2017 Jan 31;8(27):43915–24. doi: 10.18632/oncotarget.14918 (PMC5546449; doi:10.18632/oncotarget.14918)
Supplement: Supplementary file 1 [file oncotarget-08-43915-s001.pdf]

A meta-analysis of HLA peptidome composition in different hematological entities: entity-specific dividing lines and "pan-leukemia" antigens

SUPPLEMENTARY MATERIALS

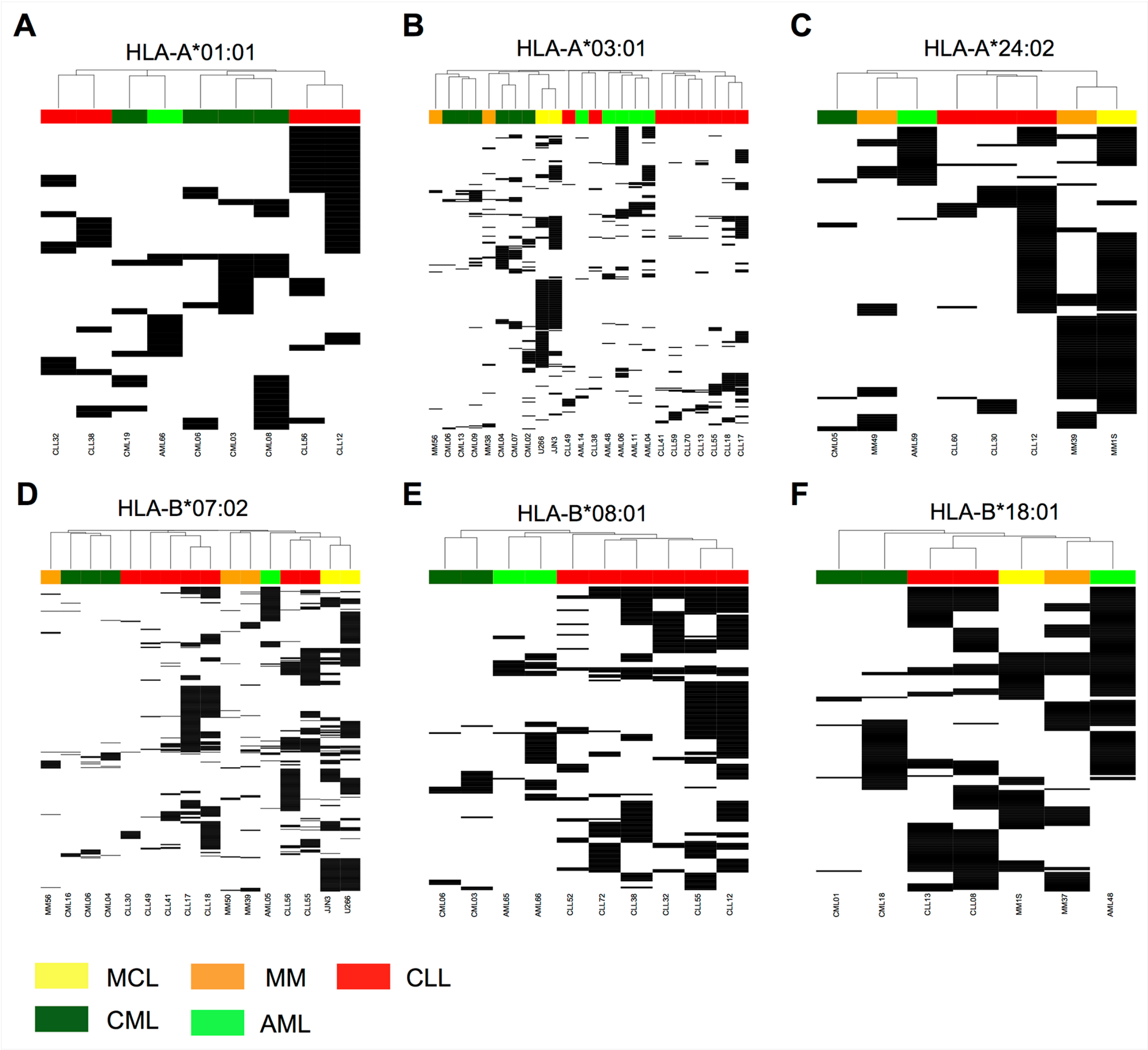

Supplementary Figure 1: HLA allotype-specific clustering analysis of "cancer-exclusive" HLA ligands on hematological cancers. A. "Cancer-exclusive" HLA-A*01:01 ligands identified on AML (light green, n=1), CML (dark green, n=4), CLL (red, n=4) and MM/MCL (orange/yellow, n=0/0) were analyzed by complete linkage clustering based on the Jaccard similarity coefficient. **B.** "Cancer-exclusive" HLA-A*03:01 ligands identified on AML (light green, n=5), CML (dark green, n=6), CLL (red, n=9) and MM/MCL (orange/yellow, n=2/2) were analyzed by complete linkage clustering based on the Jaccard similarity coefficient. **C.** "Cancer-exclusive" HLA-A*24:02 ligands identified on AML (light green, n=1), CML (dark green, n=1), CLL (red, n=3) and MM/MCL (orange/yellow, n=2/1) were analyzed by complete linkage clustering based on the Jaccard similarity coefficient. **D.** "Cancer-exclusive" HLA-B*07:02 ligands identified on AML (light green, n=1), CML (dark green, n=3), CLL (red, n=7) and MM/MCL (orange/yellow, n=3/2) were analyzed by complete linkage clusterin based on the Jaccard similarity coefficient. **E.** "Cancer-exclusive" HLA-B*08:01 ligands identified on AML (light green, n=2), CML (dark green, n=2), CLL (red, n=6) and MM/MCL (orange/yellow, n=0/0) were analyzed by complete linkage clustering based on the Jaccard similarity coefficient. **F.** "Cancer-exclusive" HLA-B*18:01 ligands identified on AML (light green, n=1), CML (dark green, n=2), CLL (red, n=2) and MM/MCL (orange/yellow, n=1/1) were analyzed by complete linkage clustering based on the Jaccard similarity coefficient.

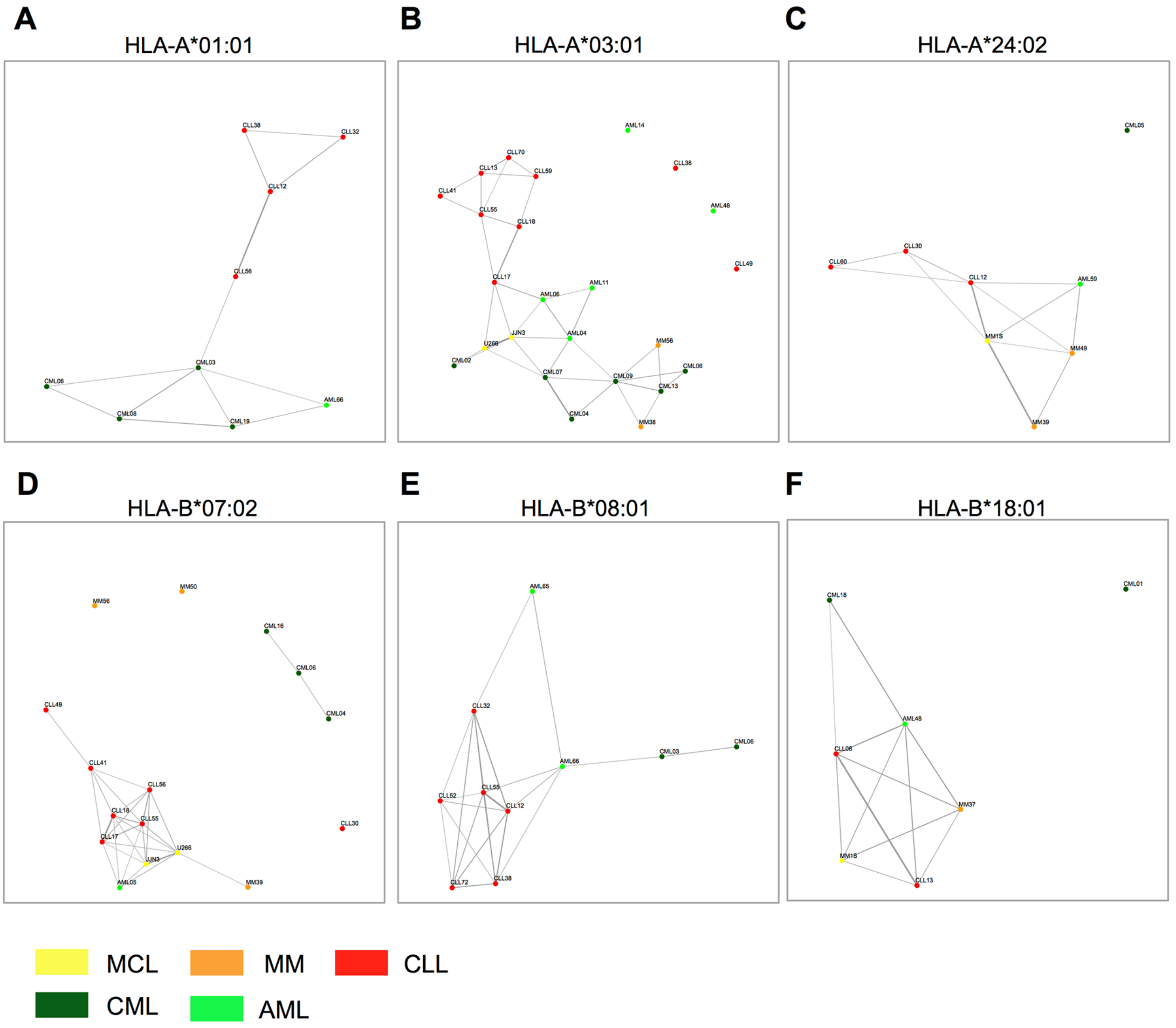

Supplementary Figure 2: HLA allotype-specific Jaccard distance graphs of "cancer-exclusive" HLA ligands on hematological cancers. Samples showing ≥10% Jaccard similiarity of their "cancer-exclusive" HLA peptidomes were linked by edges, with the thickness of the edge positively correlating with the degree of similarity. **A.** Jaccard distance graph based on "cancer-exclusive" HLA-A*01:01 ligands identified on AML (light green, n=1), CML (dark green, n=4), CLL (red, n=4) and MM/MCL (orange/yellow, n=0/0). **B.** Jaccard distance graph based on "cancer-exclusive" HLA-A*03:01 ligands identified on AML (light green, n=5), CML (dark green, n=6), CLL (red, n=9) and MM/MCL (orange/yellow, n=2/2). **C.** Jaccard distance graph based on "cancer-exclusive" HLA-A*24:02 ligands identified on AML (light green, n=1), CML (dark green, n=1), CLL (red, n=3) and MM/MCL (orange/yellow, n=2/1). **D.** Jaccard distance graph based on "cancer-exclusive" HLA-B*07:02 ligands identified on AML (light green, n=1), CML (dark green, n=3), CLL (red, n=7) and MM/MCL (orange/yellow, n=3/2). **E.** Jaccard distance graph based on "cancer-exclusive" HLA-B*08:01 ligands identified on AML (light green, n=2), CML (dark green, n=2), CLL (red, n=6) and MM/MCL (orange/yellow, gn=0/0). **F.** Jaccard distance graph based on "cancer-exclusive" HLA-B*18:01 ligands identified on AML (light green, n=1), CML (dark green, n=2), CLL (red, n=2) and MM/MCL (orange/yellow, n=1/1).

A*02 (Figure 3)

11 peptides

LLDHAPPEI (AML06)

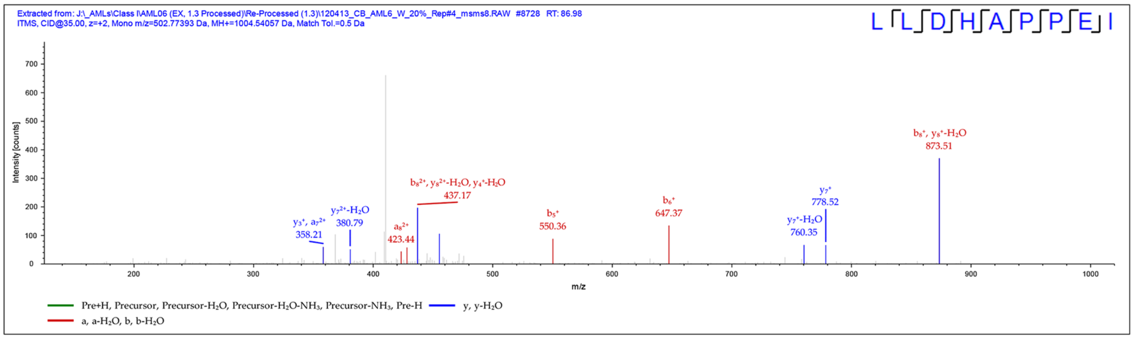

Sequence: LLDHAPPEI, Charge: +2, Monoisotopic m/z: 502.77393 Da (-0.3 mmu/-0.59 ppm), MH+: 1004.54057 Da, RT: 86.98 min,
Identified with: Mascot (v1.27); IonScore:30, Exp Value:5.2E-001, Ions matched by search engine: 8/84
Fragment match tolerance used for search: 0.5 Da
Fragments used for search: a; a-H_2O; b; b-H_2O; y; y-H_2O

Protein references (1):
- Diacylglycerol kinase zeta OS=Homo sapiens GN=DGKZ PE=1 SV=3 - [DGKZ_HUMAN]

(Continued)

ALAKLVEAI (AML05)

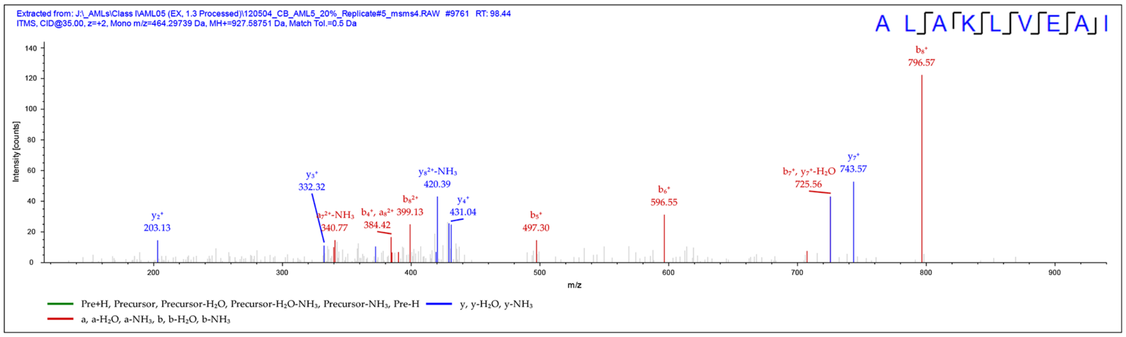

Sequence: ALAKLVEAI, Charge: +2, Monoisotopic m/z: 464.29739 Da (+0.05 mmu/+0.12 ppm), MH+: 927.58751 Da, RT: 98.44 min,
Identified with: Mascot (v1.27); IonScore:33, Exp Value:7.9E-002, Ions matched by search engine: 4/74
Fragment match tolerance used for search: 0.5 Da
Fragments used for search: a; a-H_2O; a-NH_3; b; b-H_2O; b-NH_3; y; y-H_2O; y-NH_3

Protein references (1):
- 60S ribosomal protein L7a OS=Homo sapiens GN=RPL7A PE=1 SV=2 - [RL7A_HUMAN]

ALSGLAVRL (AML05)

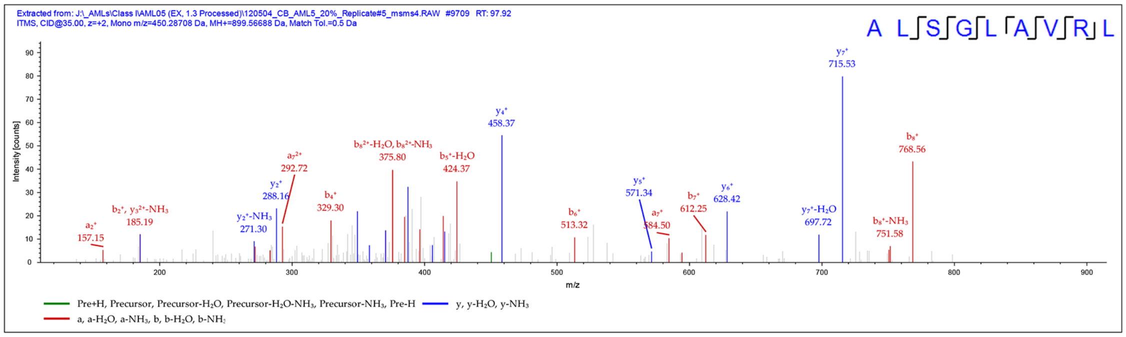

Sequence: ALSGLAVRL, Charge: +2, Monoisotopic m/z: 450.28708 Da (-0.23 mmu/-0.5 ppm), MH+: 899.56688 Da, RT: 97.92 min,
Identified with: Mascot (v1.27); IonScore:37, Exp Value:3.7E-002, Ions matched by search engine: 4/66
Fragment match tolerance used for search: 0.5 Da
Fragments used for search: a; a-H_2O; a-NH_3; b; b-H_2O; b-NH_3; y; y-H_2O; y-NH_3

Protein references (1):
- Putative uncharacterized protein CXorf69 OS=Homo sapiens GN=CXorf69 PE=2 SV=4 - [CX069_HUMAN]

(*Continued*)

GLTSTDLLFHL (AML05)

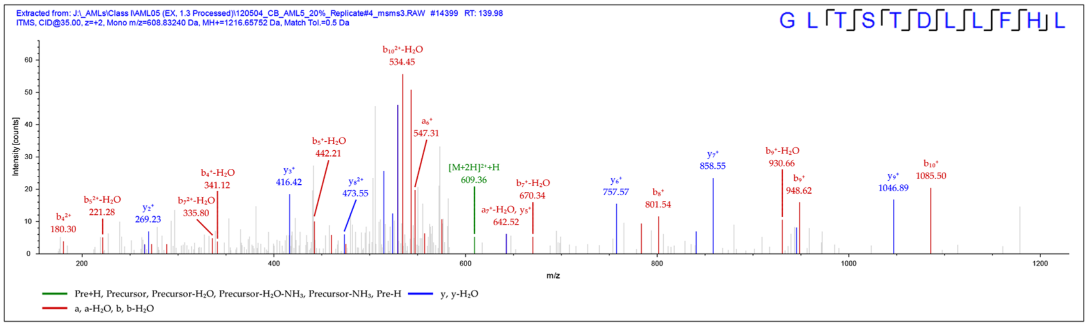

Sequence: GLTSTDLLFHL, Charge: +2, Monoisotopic m/z: 608.83240 Da (+0.13 mmu/+0.21 ppm), MH+: 1216.65752 Da, RT: 139.98 min,
Identified with: Mascot (v1.27); IonScore:22, Exp Value:3.0E+000, Ions matched by search engine: 8/104
Fragment match tolerance used for search: 0.5 Da
Fragments used for search: a; a-H_2O; b; b-H_2O; y; y-H_2O

Protein references (1):
- DNA polymerase alpha subunit B OS=Homo sapiens GN=POLA2 PE=1 SV=2 - [DPOA2_HUMAN]

VLFEGRTVQL (AML05)

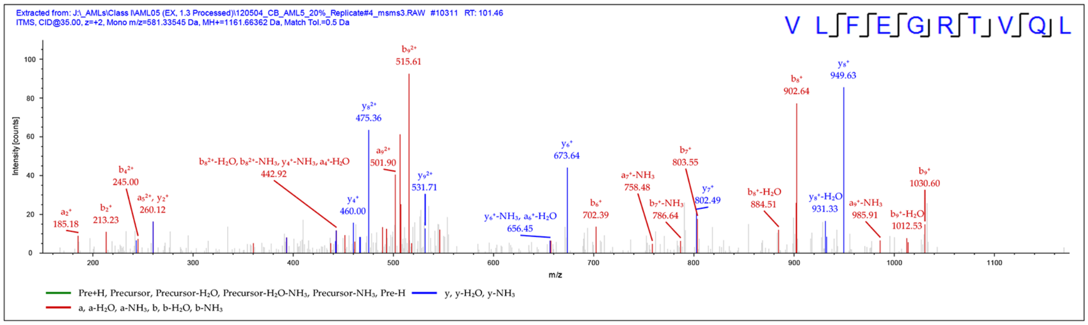

Sequence: VLFEGRTVQL, Charge: +2, Monoisotopic m/z: 581.33545 Da (+0.47 mmu/+0.8 ppm), MH+: 1161.66362 Da, RT: 101.46 min,
Identified with: Mascot (v1.27); IonScore:25, Exp Value:8.4E-001, Ions matched by search engine: 5/86
Fragment match tolerance used for search: 0.5 Da
Fragments used for search: a; a-H_2O; a-NH_3; b; b-H_2O; b-NH_3; y; y-H_2O; y-NH_3

Protein references (1):
- Cytosolic carboxypeptidase 1 OS=Homo sapiens GN=AGTPBP1 PE=1 SV=3 - [CBPC1_HUMAN]

(Continued)

FLLPIKTVGV (AML15)

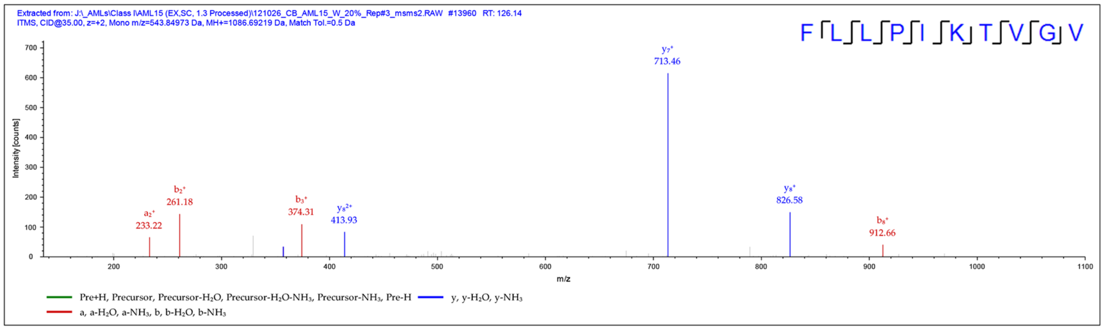

Sequence: FLLPIKTVGV, Charge: +2, Monoisotopic m/z: 543.84973 Da (-0.01 mmu/-0.01 ppm), MH+: 1086.69219 Da, RT: 126.14 min,
Identified with: Mascot (v1.27); IonScore:43, Exp Value:3.4E-003, Ions matched by search engine: 5/80
Fragment match tolerance used for search: 0.5 Da
Fragments used for search: a; a-H_2O; a-NH_3; b; b-H_2O; b-NH_3; y; y-H_2O; y-NH_3

Protein references (1):
- GMP synthase [glutamine-hydrolyzing] OS=Homo sapiens GN=GMPS PE=1 SV=1 - [GUAA_HUMAN]

SLAPLFFKL (AML15)

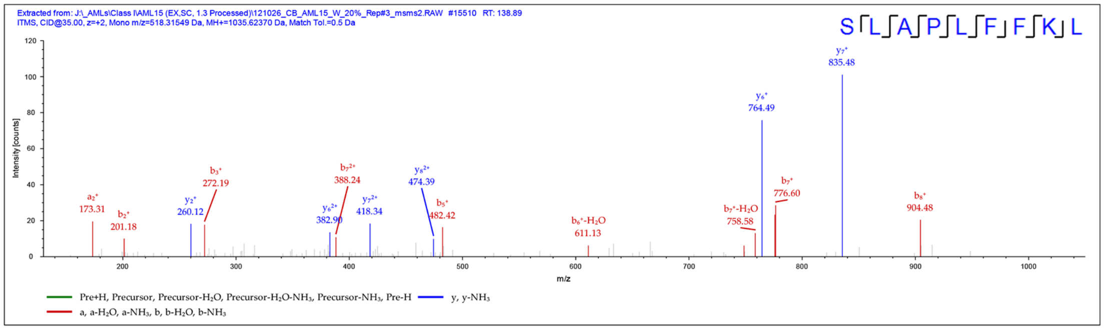

Sequence: SLAPLFFKL, Charge: +2, Monoisotopic m/z: 518.31549 Da (-0.04 mmu/-0.07 ppm), MH+: 1035.62370 Da, RT: 138.89 min,
Identified with: Mascot (v1.27); IonScore:41, Exp Value:8.4E-003, Ions matched by search engine: 6/66
Fragment match tolerance used for search: 0.5 Da
Fragments used for search: a; a-H_2O; a-NH_3; b; b-H_2O; b-NH_3; y; y-NH_3

Protein references (1):
- AP-3 complex subunit delta-1 OS=Homo sapiens GN=AP3D1 PE=1 SV=1 - [AP3D1_HUMAN]

(*Continued*)

YQIPRTFTFL (AML15)

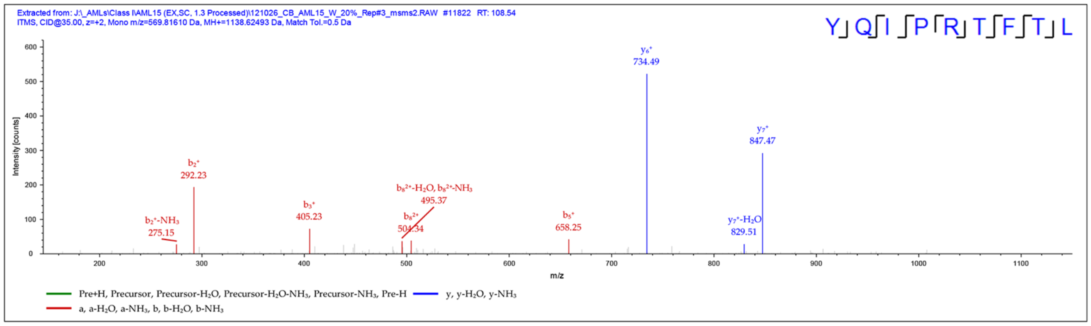

Sequence: YQIPRTFTFL, Charge: +2, Monoisotopic m/z: 569.81610 Da (-0.31 mmu/-0.55 ppm), MH+: 1138.62493 Da, RT: 108.54 min,
Identified with: Mascot (v1.27); IonScore:45, Exp Value:9.0E-003, Ions matched by search engine: 5/84
Fragment match tolerance used for search: 0.5 Da
Fragments used for search: a; a-H_2O; a-NH_3; b; b-H_2O; b-NH_3; y; y-H_2O; y-NH_3

Protein references (1):
- GRB2-associated-binding protein 2 OS=Homo sapiens GN=GAB2 PE=1 SV=1 - [GAB2_HUMAN]

SLYDYNPNL (AML37)

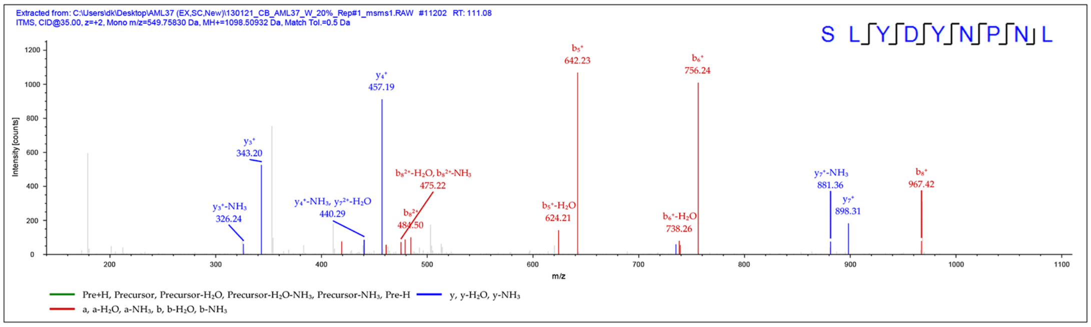

Sequence: SLYDYNPNL, Charge: +2, Monoisotopic m/z: 549.75830 Da (-0.45 mmu/-0.83 ppm), MH+: 1098.50932 Da, RT: 111.08 min,
Identified with: Mascot (v1.27); IonScore:33, Exp Value:1.6E-001, Ions matched by search engine: 10/74
Fragment match tolerance used for search: 0.5 Da
Fragments used for search: a; a-H_2O; a-NH_3; b; b-H_2O; b-NH_3; y; y-H_2O; y-NH_3

Protein references (1):
- Eukaryotic translation initiation factor 3 subunit C OS=Homo sapiens GN=EIF3C PE=1 SV=1 - [EIF3C_HUMAN]

(*Continued*)

KISEVVELL (AML37)

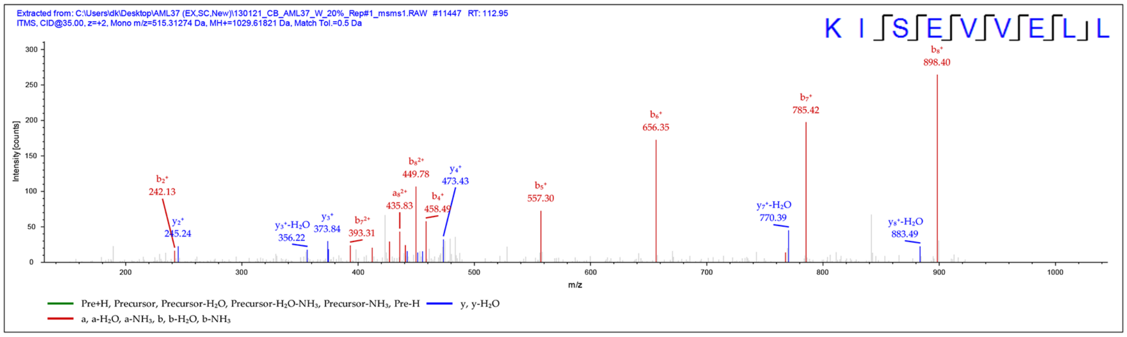

Sequence: KISEVVELL, Charge: +2, Monoisotopic m/z: 515.31281 Da (-0.38 mmu/-0.74 ppm), MH+: 1029.61833 Da, RT: 68.65 min,
Identified with: Mascot (v1.27); IonScore:20, Exp Value:1.4E+000, Ions matched by search engine: 4/80
Fragment match tolerance used for search: 0.5 Da
Fragments used for search: a; a-H_2O; a-NH_3; b; b-H_2O; b-NH_3; y; y-H_2O

Protein references (1):
- DNA mismatch repair protein Msh6 OS=Homo sapiens GN=MSH6 PE=1 SV=2 - [MSH6_HUMAN]

TLFPVRLLV (AML66)

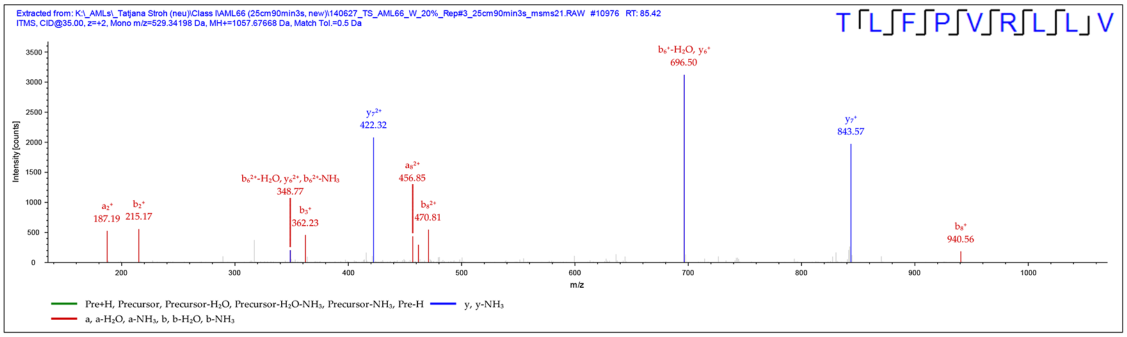

Sequence: TLFPVRLLV, Charge: +2, Monoisotopic m/z: 529.34198 Da (-0.1 mmu/-0.19 ppm), MH+: 1057.67668 Da, RT: 85.42 min,
Identified with: Mascot (v1.30); IonScore:23, Exp Value:3.0E-001, Ions matched by search engine: 11/70
Fragment match tolerance used for search: 0.5 Da
Fragments used for search: a; a-H_2O; a-NH_3; b; b-H_2O; b-NH_3; y; y-NH_3

Protein references (1):
- Lysophosphatidylcholine acyltransferase 1 OS=Homo sapiens GN=LPCAT1 PE=1 SV=2 - [PCAT1_HUMAN]

(Continued)

A*03 (suppl. Table 3)

9 peptides

GLDDPRLEK (AML04)

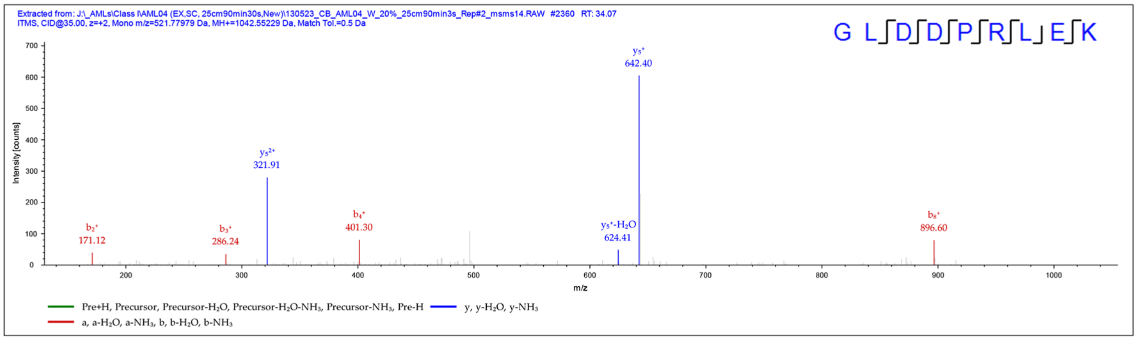

Sequence: GLDDPRLEK, Charge: +2, Monoisotopic m/z: 521.77979 Da (-0.26 mmu/-0.5 ppm), MH+: 1042.55229 Da, RT: 34.07 min,
Identified with: Mascot (v1.27); IonScore:46, Exp Value:7.8E-003, Ions matched by search engine: 6/76
Fragment match tolerance used for search: 0.5 Da
Fragments used for search: a; a-H_2O; a-NH_3; b; b-H_2O; b-NH_3; y; y-H_2O; y-NH_3

(*Continued*)

GLDPSQRPK (AML04)

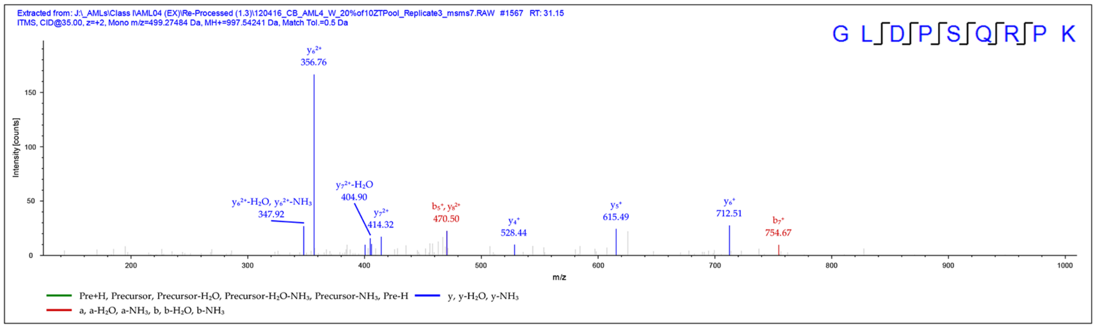

Sequence: GLDPSQRPK, Charge: +2, Monoisotopic m/z: 499.27484 Da (-0.08 mmu/-0.17 ppm), MH+: 997.54241 Da, RT: 31.15 min,
Identified with: Mascot (v1.27); IonScore:22, Exp Value:1.8E+000, Ions matched by search engine: 6/76
Fragment match tolerance used for search: 0.5 Da
Fragments used for search: a; a-H_2O; a-NH_3; b; b-H_2O; b-NH_3; y; y-H_2O; y-NH_3

Protein references (1):
- Chromosome transmission fidelity protein 18 homolog OS=Homo sapiens GN=CHTF18 PE=1 SV=1 - [CTF18_HUMAN]

KLYEKKLLKL (AML04)

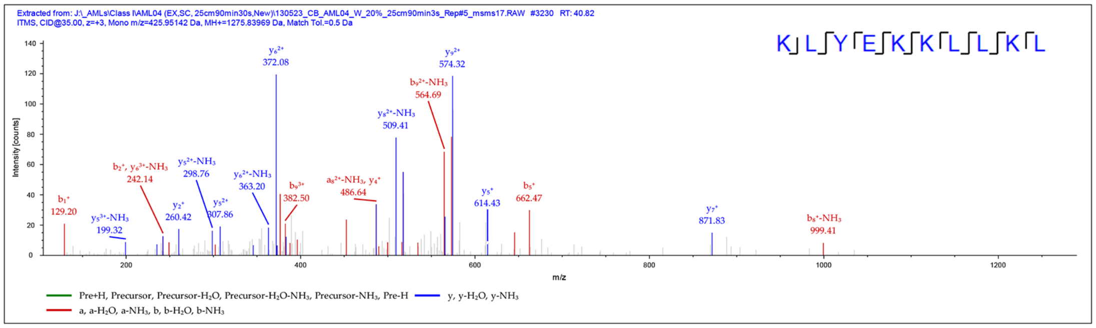

Sequence: KLYEKKLLKL, Charge: +3, Monoisotopic m/z: 425.95142 Da (-0.08 mmu/-0.18 ppm), MH+: 1275.83969 Da, RT: 40.82 min,
Identified with: Mascot (v1.27); IonScore:28, Exp Value:7.1E-003, Ions matched by search engine: 10/106
Fragment match tolerance used for search: 0.5 Da
Fragments used for search: a; a-H_2O; a-NH_3; b; b-H_2O; b-NH_3; y; y-H_2O; y-NH_3

Protein references (2):
- Lamina-associated polypeptide 2, isoform alpha OS=Homo sapiens GN=TMPO PE=1 SV=2 - [LAP2A_HUMAN]
- Lamina-associated polypeptide 2, isoforms beta/gamma OS=Homo sapiens GN=TMPO PE=1 SV=2 - [LAP2B_HUMAN]

(*Continued*)

KLYPTLVIR (AML04)

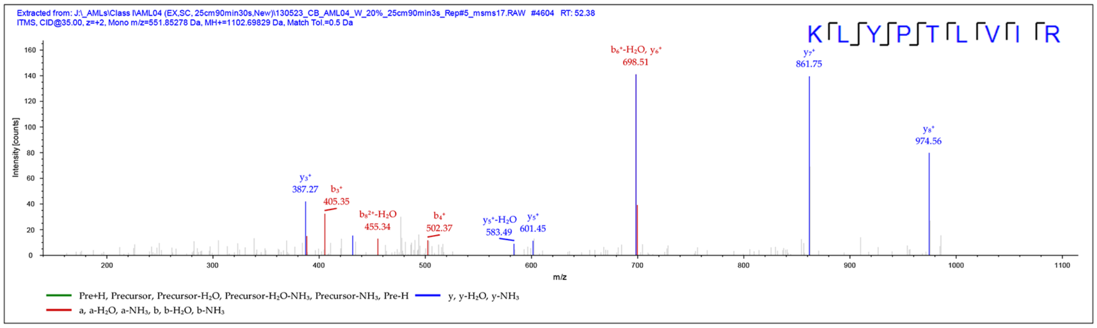

Sequence: KLYPTLVIR, Charge: +2, Monoisotopic m/z: 551.85278 Da (-0.02 mmu/-0.04 ppm), MH+: 1102.69829 Da, RT: 52.38 min,
Identified with: Mascot (v1.27); IonScore:20, Exp Value:4.1E-001, Ions matched by search engine: 4/96
Fragment match tolerance used for search: 0.5 Da
Fragments used for search: a; a-H_2O; a-NH_3; b; b-H_2O; b-NH_3; y; y-H_2O; y-NH_3

Protein references (1):
- Elongator complex protein 3 OS=Homo sapiens GN=ELP3 PE=1 SV=2 - [ELP3_HUMAN]

KMKEALLSIGK (AML04)

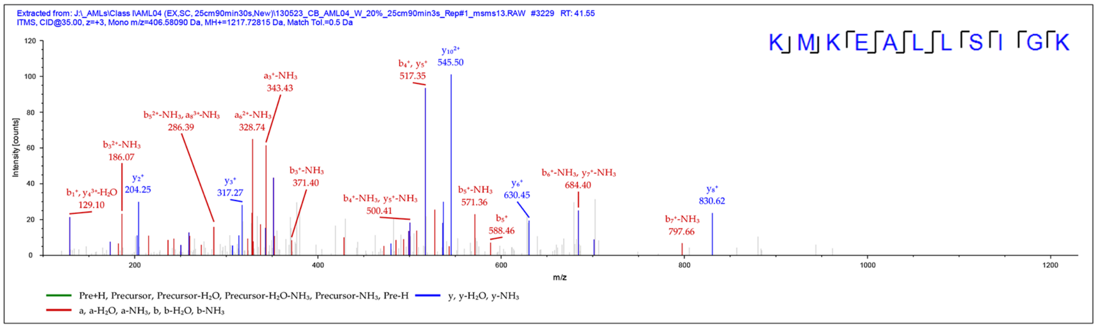

Sequence: KMKEALLSIGK, Charge: +3, Monoisotopic m/z: 406.58090 Da (-0.18 mmu/-0.43 ppm), MH+: 1217.72815 Da, RT: 41.55 min,
Identified with: Mascot (v1.27); IonScore:28, Exp Value:1.8E-001, Ions matched by search engine: 12/120
Fragment match tolerance used for search: 0.5 Da
Fragments used for search: a; a-H_2O; a-NH_3; b; b-H_2O; b-NH_3; y; y-H_2O; y-NH_3

Protein references (1):
- Protein FAM136A OS=Homo sapiens GN=FAM136A PE=1 SV=1 - [F136A_HUMAN]

(Continued)

RIAKLEAAY(AML04)

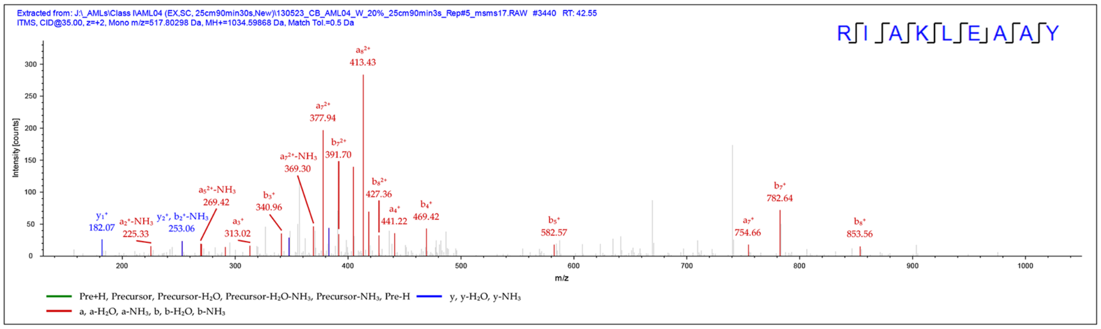

Sequence: RIAKLEAAY, Charge: +2, Monoisotopic m/z: 517.80298 Da (-0.34 mmu/-0.65 ppm), MH+: 1034.59868 Da, RT: 42.55 min,
Identified with: Mascot (v1.27); IonScore:24, Exp Value:7.9E-001, Ions matched by search engine: 7/86
Fragment match tolerance used for search: 0.5 Da
Fragments used for search: a; a-H_2O; a-NH_3; b; b-H_2O; b-NH_3; y; y-H_2O; y-NH_3

Protein references (1):
- Small subunit processome component 20 homolog OS=Homo sapiens GN=UTP20 PE=1 SV=3 - [UTP20_HUMAN]

RLMDRPIFY (AML04)

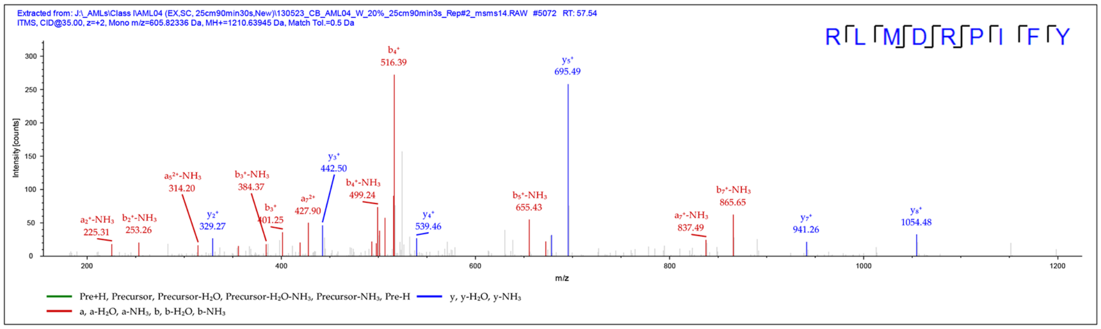

Sequence: RLMDRPIFY, Charge: +2, Monoisotopic m/z: 605.82336 Da (-0.37 mmu/-0.6 ppm), MH+: 1210.63945 Da, RT: 57.54 min,
Identified with: Mascot (v1.27); IonScore:23, Exp Value:1.5E+000, Ions matched by search engine: 8/88
Fragment match tolerance used for search: 0.5 Da
Fragments used for search: a; a-H_2O; a-NH_3; b; b-H_2O; b-NH_3; y; y-H_2O; y-NH_3

Protein references (1):
- N-acetylgalactosamine-6-sulfatase OS=Homo sapiens GN=GALNS PE=1 SV=1 - [GALNS_HUMAN]

(*Continued*)

RLNHYVLYK (AML48)

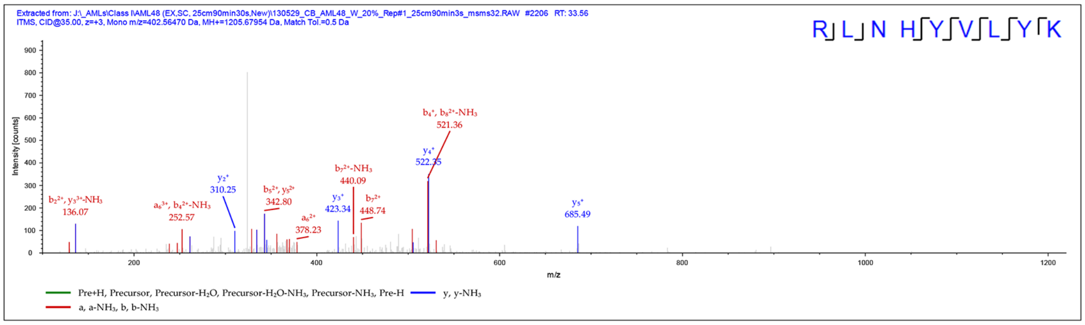

Sequence: RLNHYVLYK, Charge: +3, Monoisotopic m/z: 402.56470 Da (+0.19 mmu/+0.47 ppm), MH+: 1205.67954 Da, RT: 33.56 min,
Identified with: Mascot (v1.27); IonScore:36, Exp Value:6.2E-002, Ions matched by search engine: 10/96
Fragment match tolerance used for search: 0.5 Da
Fragments used for search: a; a-NH_3; b; b-NH_3; y; y-NH_3

Protein references (1):
- 26S proteasome non-ATPase regulatory subunit 3 OS=Homo sapiens GN=PSMD3 PE=1 SV=2 - [PSMD3_HUMAN]

RVVDGKDLTTK (AML48)

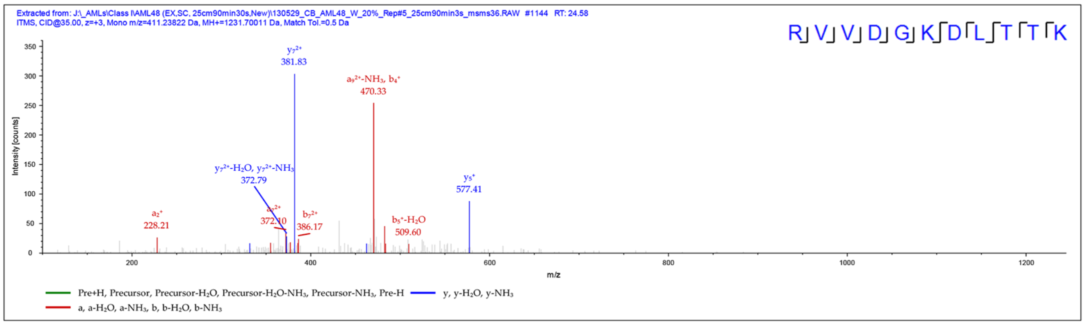

Sequence: RVVDGKDLTTK, Charge: +3, Monoisotopic m/z: 411.23822 Da (-0.14 mmu/-0.35 ppm), MH+: 1231.70011 Da, RT: 24.58 min,
Identified with: Mascot (v1.27); IonScore:10, Exp Value:2.4E+001, Ions matched by search engine: 5/120
Fragment match tolerance used for search: 0.5 Da
Fragments used for search: a; a-H_2O; a-NH_3; b; b-H_2O; b-NH_3; y; y-H_2O; y-NH_3

Protein references (1):
- Fanconi anemia group D2 protein OS=Homo sapiens GN=FANCD2 PE=1 SV=1 - [FACD2_HUMAN]

(Continued)

B*07 (suppl. Table 4)

2 peptides

APKRPPSAFF (JJN3)

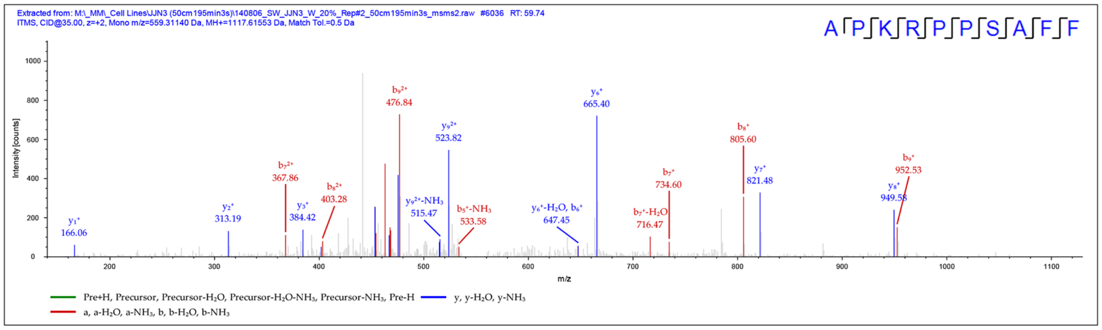

Sequence: APKRPPSAFF, Charge: +2, Monoisotopic m/z: 559.31140 Da (+0.09 mmu/+0.16 ppm), MH+: 1117.61553 Da, RT: 59.74 min,
Identified with: Mascot (v1.30); IonScore:41, Exp Value:2.2E-002, Ions matched by search engine: 6/88
Fragment match tolerance used for search: 0.5 Da
Fragments used for search: a; a-H_2O; a-NH_3; b; b-H_2O; b-NH_3; y; y-H_2O; y-NH_3

Protein references (3):
- Putative high mobility group protein B1-like 1 OS=Homo sapiens GN=HMGB1P1 PE=5 SV=1 - [HGB1A_HUMAN]
- High mobility group protein B1 OS=Homo sapiens GN=HMGB1 PE=1 SV=3 - [HMGB1_HUMAN]

(Continued)

SPIEKSGVL (JJN3)

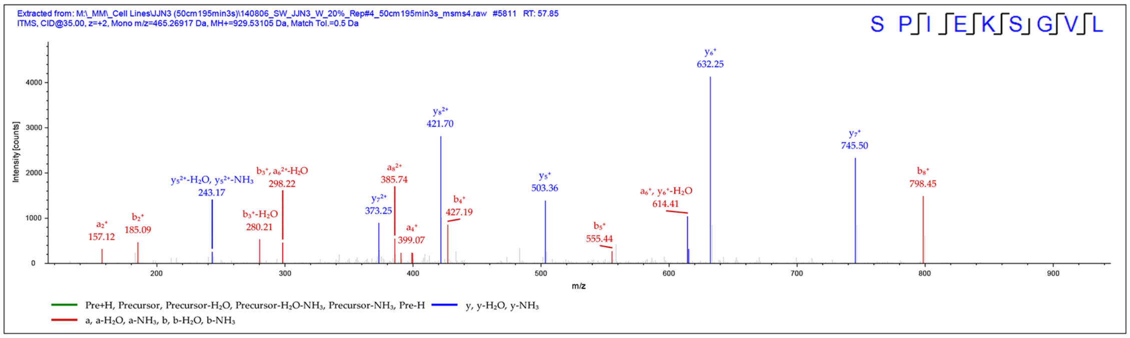

Sequence: SPIEKSGVL, Charge: +2, Monoisotopic m/z: 465.26917 Da (+0.39 mmu/+0.84 ppm), MH+: 929.53105 Da, RT: 57.85 min,
Identified with: Mascot (v1.30); IonScore:40, Exp Value:4.4E-002, Ions matched by search engine: 7/72
Fragment match tolerance used for search: 0.5 Da
Fragments used for search: a; a-H_2O; a-NH_3; b; b-H_2O; b-NH_3; y; y-H_2O; y-NH_3

Protein references (1):
- Protein CASC5 OS=Homo sapiens GN=CASC5 PE=1 SV=3 - [CASC5_HUMAN]

B*18 (suppl. Table 5)

3 peptides

(*Continued*)

DEAPPEHSF (AML48)

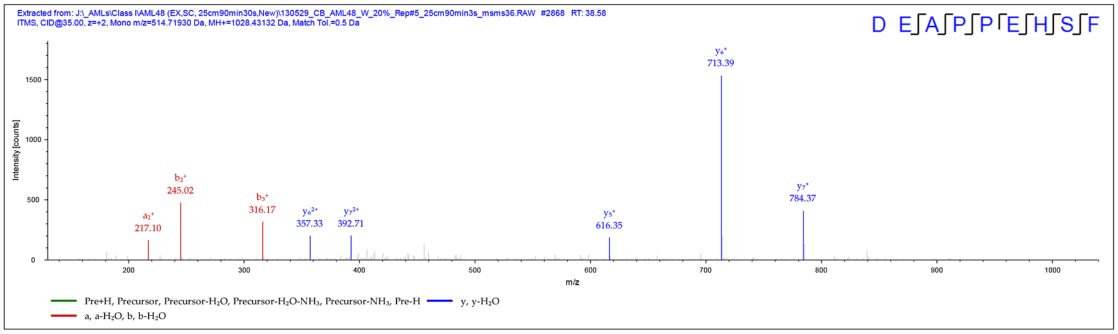

Sequence: DEAPPEHSF, Charge: +2, Monoisotopic m/z: 514.71930 Da (-0.34 mmu/-0.67 ppm), MH+: 1028.43132 Da, RT: 38.58 min,
Identified with: Mascot (v1.27); IonScore:18, Exp Value:2.5E+000, Ions matched by search engine: 3/84
Fragment match tolerance used for search: 0.5 Da
Fragments used for search: a; a-H_2O; b; b-H_2O; y; y-H_2O

Protein references (1):
- Protein phosphatase Slingshot homolog 2 OS=Homo sapiens GN=SSH2 PE=1 SV=1 - [SSH2_HUMAN]

DEHHSVNF (AML48)

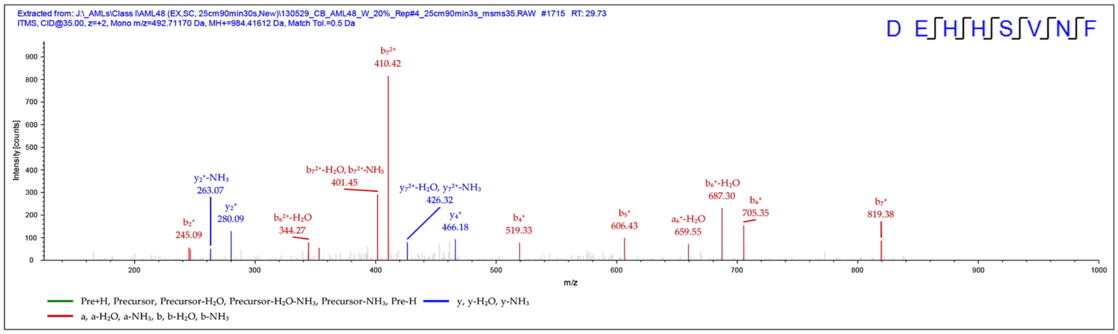

Sequence: DEHHSVNF, Charge: +2, Monoisotopic m/z: 492.71170 Da (-0.44 mmu/-0.9 ppm), MH+: 984.41612 Da, RT: 29.73 min,
Identified with: Mascot (v1.27); IonScore:24, Exp Value:6.1E-001, Ions matched by search engine: 5/58
Fragment match tolerance used for search: 0.5 Da
Fragments used for search: a; a-H_2O; a-NH_3; b; b-H_2O; b-NH_3; y; y-H_2O; y-NH_3

Protein references (1):
- Telomere-associated protein RIF1 OS=Homo sapiens GN=RIF1 PE=1 SV=2 - [RIF1_HUMAN]

(*Continued*)

DETSALKF (AML48)

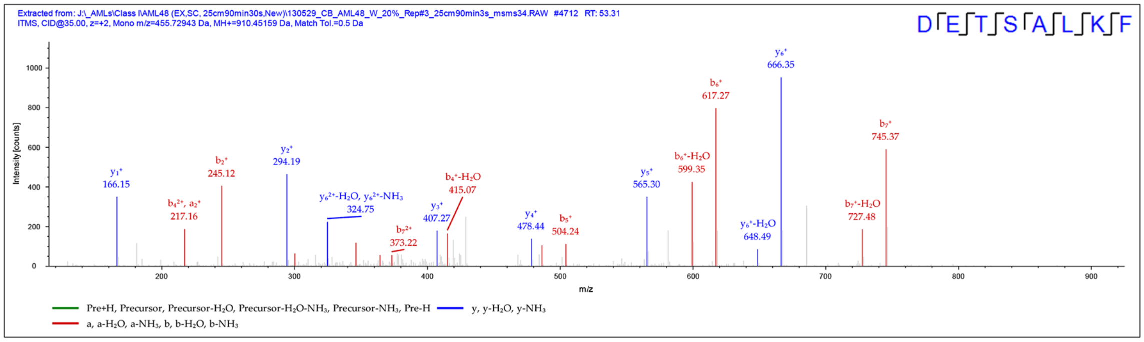

Sequence: DETSALKF, Charge: +2, Monoisotopic m/z: 455.72943 Da (-0.05 mmu/-0.11 ppm), MH+: 910.45159 Da, RT: 53.31 min,
Identified with: Mascot (v1.27); IonScore:40, Exp Value:3.0E-002, Ions matched by search engine: 7/58
Fragment match tolerance used for search: 0.5 Da
Fragments used for search: a; a-H_2O; a-NH_3; b; b-H_2O; b-NH_3; y; y-H_2O; y-NH_3

Protein references (1):
- Integrin alpha-4 OS=Homo sapiens GN=ITGA4 PE=1 SV=3 - [ITA4_HUMAN]

Supplementary Figure 3: LTQ-MS2 fragment spectra of "pan-leukemia" peptides. Representative LTQ-MS2 fragment spectra of the 25 "pan-leukemia" peptides (11 A*02:01, 9 A*03:01, 2 B*07:02, 3 B*18:01) identified on different hematological malignancies.

Supplementary Table 1: Tissue samples and peptide yields comprised in the non-malignant primary tissue database

Tissue	Number of analyzed samples	Unique peptide IDs
Brain	5	2208
Kidney	30	16045
Lung	3	5427
Muscle	2	583
Small Intestine	2	3098
Spleen	2	4585
Bladder	1	1416
Heart	1	1128
Myelon	1	383
Pancreas	2	1576
Skin	2	543
Stomach	1	1198
Thyroid	1	1451
Adrenal Gland	1	690
Esophagus	1	392
Liver	13	10081
Testicle	1	1736
Trachea	1	334
Bone Marrow	9	3591
PBMC	30	17322
Granulocytes	3	4224
Colon	32	12539
Ovary	3	1036

PBMC = peripheral mononuclear blood cells.

Supplementary Table 2: "Cancer-exclusive" & overall HLA ligand IDs on hematological malignancies.

See Supplementary File 1

Supplementary Table 3: Presentation of "cancer-exclusive" HLA-A*03:01 ligands across different hematological malignancies

HLA ligand	Source protein	Number of positive hematological malignancies (Frequency [%])			
		AML (n=5)	CML (n=6)	CLL (n=9)	MM/MCL (n=2/2)
GLDDPRLEK	LRPAP1	3 (60.0)	3 (50.0)	1 (11.1)	1 (25.0)
GLDPSQRPK	CHTF18	2 (40.0)	1 (16.7)	3 (33.3)	2 (50.0)
KLYEKKLLKL	TMPO	1 (20.0)	1 (16.7)	3 (33.3)	2 (50.0)
KLYPTLVIR	ELP3	4 (80.0)	1 (16.7)	4 (44.4)	1 (25.0)
KMKEALLSIGK	FAM136A	2 (40.0)	1 (16.7)	1 (11.1)	2 (50.0)
RIAKLEAAY	UTP20	1 (20.0)	2 (33.3)	1 (11.1)	2 (50.0)
RLMDRPIFY	GALNS	1 (20.0)	2 (33.3)	1 (11.1)	1 (25.0)
RLNHYVLYK	PSMD3	2 (40.0)	1 (16.7)	2 (22.2)	2 (50.0)
RVVDGKDLTTK	FANCD2	1 (20.0)	1 (16.7)	4 (44.4)	2 (50.0)

HLA-A*03:01 restricted "pan-leukemia" antigens identified across all four hematological malignancies. Peptides represented with frequencies ≥20% across all entities are marked in dark red, peptides represented with frequencies ≥20% across entities of the same lineage are marked in light red. A minimum value of n≥4 allotype positive samples was required for the calculation of presentation frequencies.

Supplementary Table 4: Presentation of "cancer-exclusive" HLA-B*07:02 ligands across different hematological malignancies

HLA ligand	Source protein	Number of positive hematological malignancies (Frequency [%])			
		AML (n=1)	CML (n=3)	CLL (n=7)	MM/MCL (n=3/2)
APKRPPSAFF	HMGB1P1, HMGB1, HMGB2	1 (100.0)	1 (33.3)	2 (28.6)	2 (40.0)
SPIEKSGVL	CASC5	1 (100.0)	1 (33.3)	1 (14.3)	2 (40.0)

HLA-B*07:02 restricted "pan-leukemia" antigens identified across all four hematological malignancies. Peptides represented with frequencies ≥20% across all entities are marked in dark red, peptides represented with frequencies ≥20% across entities of the same lineage are marked in light red. A minimum value of n≥4 allotype positive samples was required for the calculation of presentation frequencies.

Supplementary Table 5: Presentation of "cancer-exclusive" HLA-B*18:01 ligands across different hematological malignancies

HLA ligand	Source protein	Number of positive hematological malignancies (Frequency [%])			
		AML (n=1)	CML (n=2)	CLL (n=2)	MM/MCL (n=1/1)
DEAPPEHSF	DGKZ	1 (100.0)	1 (50.0)	2 (100.0)	2 (100.0)
DEHHSVNF	RPL7A	1 (100.0)	1 (50.0)	2 (100.0)	2 (100.0)
DETSALKF	SMIM10	1 (100.0)	1 (50.0)	1 (50.0)	1 (50.0)

HLA-B*18:01 restricted „pan-leukemia“ antigens identified across all four hematological malignancies. Peptides represented with frequencies ≥20% across all entities are marked in dark red, peptides represented with frequencies ≥20% across entities of the same lineage are marked in light red. A minimum value of n≥4 allotype positive samples was required for the calculation of presentation frequencies.
