# Supplementary material for: A meta-analysis of HLA peptidome composition in different hematological entities: entity-specific dividing lines and “pan-leukemia” antigens
Source: Oncotarget. 2017 Jan 31;8(27):43915–24. doi: 10.18632/oncotarget.14918 (PMC5546449; doi:10.18632/oncotarget.14918)
Supplement: Supplementary file 2 [file oncotarget-08-43915-s002.docx]

**Supplementary Table 2:**

“Cancer-exclusive” & overall HLA ligand IDs on hematological malignancies

| **Sample** | **HLA typing** | **Sample**  **[10^9^ cells]** | **A*01:01** | **A*02:01** | **A*03:01** | **A*24:02** | **B*07:02** | **B*08:01** | **B*18:01** |
| --- | --- | --- | --- | --- | --- | --- | --- | --- | --- |
| AML01 | A*02, A*11, B*35, B*44 | 0.9 | - | 4 (57) | - | - | - | - | - |
| AML04 | A*03:01, B*39:01, B*51:01 | 1.0 | - | - | 87 (438) | - | - | - | - |
| AML05 | A*02, B*07, B*40 | 0.4 | - | 263 (641) | - | - | 139 (447) | - | - |
| AML06 | A*02, A*03, B*44:25, B*52:15, | 1.6 | - | 19 (164) | 118 (535) | - | - | - | - |
| AML11 | A*02:01, A*03:01, B*38:01, B*44:02, | 2.8 | - | 28 (235) | 41 (373) | - | - | - | - |
| AML14 | A*03:01, A*26:01, B*35:01, B*38:01, | 1.7 | - | - | 11 (211) | - | - | - | - |
| AML15 | A*02, A*66, B*40 , B*15, | 8.4 | - | 112 (560) | - | - | - | - | - |
| AML36 | A*02:01, A*23:01, B*44:02 , B*49:01 | 0.4 | - | 23 (208) | - | - | - | - | - |
| AML37 | A*02:01, B*13:02, B*51:01 | 0.2 | - | 122 (610) | - | - | - | - | - |
| AML48 | A*02:01, A*03:01, B*18:01 | 2.6 | - | 10 (153) | 19 (218) | - | - | - | 212 (511) |
| AML49 | A*02, A*26:01, B*27:05 | 4.9 | - | 1 (28) | - | - | - | - | - |
| AML59 | A*02, A*24, B*44, B*50 | 19.0 | - | 27 (29) | - | 45 (294) | - | - | - |
| AML64 | A*01:01, A*23:01, B*44:03 | 0.5 | 10 (175) | - | - | - | - | - | - |
| AML65 | A*02:01, A*11:01, B*08:01, B*57:01 | 0.5 | - | 11 (209) | - | - | - | 15 (154) | - |
| AML66 | A*01, A*02, B*08, B*13 | 0.5 | 34 (255) | 132 (600) | - | - | - | 115 (322) | - |
| AML70 | A*03:01, A*32:01, B*57:01, B*35:01 | 0.5 | - | - | 6 (143) | - | - | - | - |
| CML01 | A*23, A*25, B*18, B*57 | 0.9 | - | - | - | - | - | - | 11 (81) |
| CML02 | A*03, B*35, B*52 | 4.8 | - | - | 127 (691) | - | - | - | - |
| CML03 | A*01, B*08 | 0.4 | 22 (228) | - | - | - | - | 40 (132) | - |
| CML04 | A*03, A*68, B*07, B*44 | 5.0 | - | - | 55 (294) | - | 30 (216) | - | - |
| CML05 | A*24, A*28, B*27, B*57 | 2.9 | - | - | - | 11 (148) | - | - | - |
| CML06 | A*01, A*03, B*07, B*08 | 5.0 | 8 (103) | - | 17 (145) | - | 27 (205) | 19 (143) | - |
| CML07 | A*02, A*03, B*13, B*15 | 5 | - | 58 (382) | 63 (387) | - | - | - | - |
| CML08 | A*01, A*02, B*08, B*50 | 6 | 33 (212) | 43 (165) | - | - | - | 17 (72) | - |
| CML09 | A*03, B*07, B*35 | 1,3 | - | - | 32 (287) | - | 19 (170) | - | - |
| CML10 | A*02, A*11, B*35, B*44 | 0.5 | - | 159 (570) | - | - | - | - | - |
| CML13 | A*02:01, A*03:01, B*07:01, B*51:01 | 2.0 | - | 50 (340) | 34 (364) | - | 0 (0) | - | - |
| CML15 | A*01:01, A*02:01, B*51:01 | 0.5 | 8 (86) | 7 (118) | - | - | - | - | - |
| CML16 | A*02:01, A*11:01, B*07 | 0.5 | - | 20 (197) | - | - | 20 (141) | - | - |
| CML18 | A*02, B*18, B*57 | 17 | - | 76 (396) | - | - | - | - | 67 (217) |
| CML19 | A*01:01, B*15:03 | 7.6 | 29 (132) | - | - | - | - | - | - |
| CLL02 | A*02, A*11, B*39, B*40 | 20.0 | - | 22 (154) | - | - | - | - | - |
| CLL04 | A*02:01, B*35:01, B*39:01 | 6.2 | - | 30 (207) | - | - | - | - | - |
| CLL08 | A*25, A*26, B*18, B*38 | 1.8 | - | - | - | - | - | - | 184 (487) |
| CLL09 | A*02, B*55, B*57 | 2.4 | - | 15 (109) | - | - | - | - | 0 (0) |
| CLL10 | A*02, A*23, B*15, B*41 | 5.8 | - | 32 (269) | - | - | - | - | - |
| CLL12 | A*01, A*24, B*08, B*27 | 11.5 | 104 (484) | - | - | 159 (555) | - | 244 (534) | - |
| CLL13 | A*02, A*03, B*18, B*35 | 5.2 | - | 12 (154) | 30 (322) | - | - | - | 107 (332) |
| CLL16 | A*24, A*31, B*15, B*38 | 6.4 | - | - | - | 0 (33) | - | - | - |
| CLL17 | A*03, A*30, B*07, B*13 | 0.8 | - | - | 231 (764) | - | 269 (772) | - | - |
| CLL18 | A*02, A*03, B*07, B*55 | 2.6 | - | 55 (294) | 50 (390) | - | 344 (983) | - | - |
| CLL20 | A*01, A*02, B*27, B*37 | 0.5 | 8 (132) | 19 (192) | - | - | - | - | - |
|  |  |  |  |  |  |  |  |  |  |
| CLL21 | A*02, A*68, B*15, B*27 | 3.6 | - | 88 (474) | - | - | - | - | - |
| CLL27 | A*02, A*03, B*35, B*57 | 0.9 | - | 2 (80) | 3 (93) | - | - | - | - |
| CLL28 | A*02, B*15, B*44 | 2.8 | - | 22 (266) | - | - | - | - | - |
| CLL30 | A*24, B*07, B*49:01 | 2.4 | - | - | - | 26 (161) | 36 (177) | - | - |
| CLL32 | A*01, A*68, B*08, B*44 | 2.0 | 12 (197) | - | - | - | - | 57 (193) | - |
| CLL34 | A*02, A*03, B*40 | 1.4 | - | 21 (169) | 4 (171) | - | - | - | - |
| CLL37 | A*02, A*11, B*35, B*37 | 0.4 | - | 0 (24) | - | - | - | - | - |
| CLL38 | A*01, A*03, B*08, B*51 | 0.8 | 14 (193) | - | 12 (282) | - | - | 114 (293) | - |
| CLL41 | A*02, A*03, B*07, B*44 | 1.0 | - | 9 (120) | 8 (196) | - | 56 (401) | - | - |
| CLL43 | A*24, B*35, B*50 | 1.5 | - | - | - | 2 (90) | - | - | - |
| CLL47 | A*24, A*32, B*27, B*51 | 1.5 | - | - | - | 7 (91) | - | - | - |
| CLL49 | A*03, B*07 | 1.2 | - | - | 34 (218) | - | 53 (216) | - | - |
| CLL52 | A*01, A*02, B*08, B*13 | 0.9 | 9 (183) | 70 (369) | - | - | - | 39 (211) | - |
| CLL55 | A*03, A*26, B*07, B*08 | 2.0 | - | - | 36 (325) | - | 221 (791) | 233 (665) | - |
| CLL56 | A*01, A*32, B*07, B*44 | 120.0 | 75 (277) | - | - | - | 260 (759) | - | - |
| CLL59 | A*02, A*03, B*35, B*40 | 3.9 | - | 9 (122) | 26 (304) | - | - | - | - |
| CLL60 | A*02, A*24, B*51, B*57 | 1.9 | - | 7 (104) | - | 17 (189) | - | - | - |
| CLL70 | A*02, A*03, B*40, B*44 | 0.2 | - | 29 (280) | 8 (186) | - | - | - | - |
| CLL71 | A*02, A*11, B*35 | 0.5 | - | 33 (179) | - | - | - | - | - |
| CLL72 | A*02, B*08, B*51 | 0.5 | - | 30 (292) | - | - | - | 60 (273) | - |
| CLL80 | A*02, A*24, B*51 | 0.2 | - | 9 (79) | - | 3 (76) | - | - | - |
| CLL84 | A*02, B*40, B*51 | 1 | - | 7 (140) | - | - | - | - | - |
| MM34 | A*01, A*24, B*08, B*18 |  | 2 (72) | - | - | 0 (34) | - | 1 (7) | 3 (92) |
| MM36 | A*01, A*02, B*08, B*37 | 1.2 | 12 (106) | 9 (143) | - | - | - | 7 (57) | - |
| MM37 | A*02, A*33, B*15, B*18 | 1.8 | - | 27 (248) | - | - | - | - | 120 (542) |
| MM38 | A*03, A*26, B*40, B*55 | 1.2 | - | - | 39 (362) | - | - | - | - |
| MM39 | A*02, A*24, B*07, B*27 | 4.5 | - | 32 (275) | - | 129 (360) | 74 (362) | - | - |
| MM40 | A*02,A*03, B*07, B*35 | 0.1 | - | 1 (48) | 1 (69) | - | 9 (122) | - | - |
| MM49 | A*24, A*25, B*39, B*40 | 1.3 | - | - | - | 49 (347) | - | - | - |
| MM50 | A*02, B*07, B*44 | 0.5 | - | 54 (541) | - | - | 26 (285) | - | - |
| MM56 | A*03, A*33, B*07 | 0.4 | - | - | 12 (128) | - | 59 (310) | - | - |
| MM1S | A*23:01, A*24:02, B*18:01, B*42:01 | 2 | - | - | - | 494 (953) | - | - | 171 (372) |
| U266 | A*02:01, A*03:01, B*07:02, B*40:01 | 2 | - | 246 (573) | 260 (657) | - | 581 (1084) | - | - |
| JJN3 | A*03:01, A*33:01, B*07:02, B*14:02 | 2 | - | - | 295 (810) | - | 308 (750) | - | - |

“Cancer-exclusive” peptides (overall HLA ligand IDs) identified from AML (n=16), CML (n=15), CLL (n=33) and MM/MCL (n=9/3) samples were annotated using NetMHCpan 3.0 and their respective sources’ HLA type and only binding peptides (netMHC IC_50_≤500 nM and/or percentile rank ≤2%) were retained for further analysis. Only samples expressing at least one of the 7 major HLA allotypes (A*01:01, A*02:01, A*03:01, A*24:02, B*07:02, B*08:01, B*18:01) are listed. For clustering, Jaccard distance graphs and overlap analysis of tumor-exclusive HLA ligand datasets, HLA ligands occurring only once across all samples were discarded and only samples containing ≥5 unique HLA class I ligands were included.
